# p-tau Ser356 is associated with Alzheimer’s disease pathology and is lowered in brain slice cultures using the NUAK inhibitor WZ4003

**DOI:** 10.1007/s00401-023-02667-w

**Published:** 2024-01-04

**Authors:** Lewis W. Taylor, Elizabeth M. Simzer, Claire Pimblett, Oscar T. T. Lacey-Solymar, Robert I. McGeachan, Soraya Meftah, Jamie L. Rose, Maxwell P. Spires-Jones, Kristján Holt, James H. Catterson, Henner Koch, Imran Liaquat, Jonathan H. Clarke, John Skidmore, Colin Smith, Sam A. Booker, Paul M. Brennan, Tara L. Spires-Jones, Claire S. Durrant

**Affiliations:** 1 Centre for Discovery Brain Sciences, The University of Edinburgh, Edinburgh, UK; 2 UK Dementia Research Institute, The University of Edinburgh, Edinburgh, UK; 3 The Hospital for Small Animals, Royal (Dick) School of Veterinary Studies, The University of Edinburgh, Edinburgh, UK; 4 Department of Neurology, Epileptology, RWTH Aachen University Hospital, 52074 Aachen, Germany; 5 Department of Clinical Neuroscience, Royal Infirmary of Edinburgh, 51 Little France Crescent, Edinburgh, UK; 6 The ALBORADA Drug Discovery Institute, University of Cambridge, Island Research Building, Cambridge Biomedical Campus, Hills Road, Cambridge, UK; 7 The Centre for Clinical Brain Sciences, The University of Edinburgh, Edinburgh, UK; 8 Simons Initiative for the Developing Brain, The University of Edinburgh, Edinburgh, UK; 9 Cancer Research UK Brain Tumour Centre of Excellence, CRUK Edinburgh Centre, The University of Edinburgh, Edinburgh, UK

**Keywords:** Tau, p-tau Ser356, NUAK1, Alzheimer’s disease, Mouse organotypic hippocampal slice cultures, Human brain slice cultures

## Abstract

Tau hyperphosphorylation and aggregation is a common feature of many dementia-causing neurodegenerative diseases. Tau can be phosphorylated at up to 85 different sites, and there is increasing interest in whether tau phosphorylation at specific epitopes, by specific kinases, plays an important role in disease progression. The AMP-activated protein kinase (AMPK)-related enzyme NUAK1 has been identified as a potential mediator of tau pathology, whereby NUAK1-mediated phosphorylation of tau at Ser356 prevents the degradation of tau by the proteasome, further exacerbating tau hyperphosphorylation and accumulation. This study provides a detailed characterisation of the association of p-tau Ser356 with progression of Alzheimer’s disease pathology, identifying a Braak stage-dependent increase in p-tau Ser356 protein levels and an almost ubiquitous presence in neurofibrillary tangles. We also demonstrate, using sub-diffraction-limit resolution array tomography imaging, that p-tau Ser356 co-localises with synapses in AD postmortem brain tissue, increasing evidence that this form of tau may play important roles in AD progression. To assess the potential impacts of pharmacological NUAK inhibition in an ex vivo system that retains multiple cell types and brain-relevant neuronal architecture, we treated postnatal mouse organotypic brain slice cultures from wildtype or APP/PS1 littermates with the commercially available NUAK1/2 inhibitor WZ4003. Whilst there were no genotype-specific effects, we found that WZ4003 results in a culture-phase-dependent loss of total tau and p-tau Ser356, which corresponds with a reduction in neuronal and synaptic proteins. By contrast, application of WZ4003 to live human brain slice cultures results in a specific lowering of p-tau Ser356, alongside increased neuronal tubulin protein. This work identifies differential responses of postnatal mouse organotypic brain slice cultures and adult human brain slice cultures to NUAK1 inhibition that will be important to consider in future work developing tau-targeting therapeutics for human disease.

**Supplementary Information:**

The online version contains supplementary material available at 10.1007/s00401-023-02667-w.

## Introduction

Hyperphosphorylation and aggregation of tau is a key feature of a number of dementia-causing diseases. These include both primary tauopathies, such as progressive supranuclear palsy (PSP), corticobasal degeneration (CBD) and frontotemporal dementias (FTD) with tau pathology, and secondary tauopathies, such as Alzheimer’s disease (AD), where accumulation of amyloid-beta (Aβ) is thought to initiate the disease cascade [30, 63]. Whilst tau plays a number of important physiological roles in the brain, including regulating microtubule function, myelination, neuronal excitability and DNA protection [34], there is strong evidence that hyperphosphorylated, oligomeric tau disrupts synaptic function and may be an important driver of synapse loss and neurodegeneration in dementia [4, 58]. Tau can be phosphorylated at up to 85 different sites (45 serine, 35 threonine and 5 tyrosine residues), with the levels of phosphorylation regulated by an ever expanding list of kinases and phosphatases [25, 35, 66]. There is increasing focus on whether specific forms of phosphorylated tau are key drivers of downstream pathology, and whether targeting upstream kinases could be an effective therapeutic tool to mitigate tau pathology in dementia [37, 45, 66].

Of recent interest is the possibility that increased activity of the AMP-activated protein kinase (AMPK)-related kinase, NUAK1, in AD and primary tauopathies, results in the specific phosphorylation of tau at Ser356 [37]. Ser356 is located in repeat 4 of the microtubule binding domains, so phosphorylation of this site is likely to disrupt key aspects of physiological tau function [26]. Interestingly, studies in *Drosophila melanogaster* have highlighted that phosphorylation of tau at Ser356 could be an important catalyst for further downstream phosphorylation and aggregation [2, 46]. NUAK1-mediated phosphorylation of tau at Ser356 has also been identified as a mechanism for regulating *total* tau levels, with the chaperone C-terminus of Hsc70-interacting protein (CHIP) unable to bind tau phosphorylated at Ser356, thus preventing ubiquitination of tau and its subsequent degradation by the proteasome [16, 17, 37]. Previous work reports that NUAK1 protein expression is increased in postmortem AD and PSP middle frontal gyrus and is found to co-localise with neurofibrillary tangles [37]. By crossing NUAK1^+/−^ mice to the tauopathy P301S mouse model, Lasagna-Reeves et al. found that reduction of NUAK1 lowered both p-tau Ser356 and total tau levels and rescued aspects of tau pathology [37]. This work highlighted NUAK1 as an attractive target for therapeutic development in primary tauopathies, opening important questions about whether similar strategies could be applicable to secondary tauopathies such as AD.

Whilst there are reports that NUAK1 levels may be upregulated [37] and tau is phosphorylated at Ser356 in end-stage AD [3, 18, 26, 27, 64], the progression of p-tau Ser356 accumulation over the disease time course, its representation in tangles and its association with synapses (synaptic tau has been found to be important for both tau toxicity and trans-synaptic tau spread [9, 36, 47, 68]) have not been fully characterised. Of the few studies that do examine appearance of this epitope in AD brain tissue, many use the 12E8 antibody [21, 60], which shows considerable preference for p-tau Ser262, complicating interpretation of the unique involvement of p-tau Ser356 [55]_._ In this work, we characterise specific accumulation of p-tau Ser356 in AD brain using biochemical and histological methods including sub-diffraction limit resolution microscopy to examine synapses [9, 33, 47, 52].

Based on the likely pathological involvement of p-tau Ser356, we further explore the effects of pharmacological inhibition of phosphorylation of tau at this residue. We characterise the impact of the commercially available NUAK inhibitor WZ4003, which has been previously shown to inhibit NUAK1 activity in vitro [5] and reduce p-tau Ser356 in neuroblastoma cells [37]. In this study, we look to examine the impact of WZ4003 treatment under a number of physiological and pathological conditions using both mouse and human organotypic brain slice cultures, which retain physiologically relevant neuronal architecture, supporting cell types and synaptic connections for several weeks in vitro [12, 19, 20, 28, 56]*.* We explore the impact of WZ4003 in both wildtype and APP/PS1 cultures, to assess whether NUAK1 inhibition has differential impacts on models of Aβ pathology, partially replicating changes seen in Alzheimer’s disease, where dysregulated Aβ processing is thought to drive downstream changes to tau [63]. Our results reinforce the importance of p-tau Ser356 in AD and highlight potential biological differences in mouse and human brain in terms of how NUAK1 regulates tau turnover.

## Materials and methods

### Human postmortem brain tissue

All postmortem brain tissue used in this study was obtained with ethical approval from the Edinburgh Sudden Death Brain Bank. This study was reviewed and approved by the Edinburgh Brain Bank ethics committee, a joint office for NHS Lothian and the University of Edinburgh, under ethical approval number 15-HV-016. The Edinburgh Brain Bank is a Medical Research Council funded facility with research ethics committee (REC) approval (16/ES/0084). Individual demographics/ case numbers for human postmortem studies are listed in Table 1**,** with the specific experiment they were included in referenced by the figure numbers in the table**.** Different cases were used for different experiments in line with tissue availability and type required (e.g. frozen, paraffin sections). Types of tissue used and grouped demographic information are detailed in the methods sections below for each experiment. All cases were screened by Edinburgh MRC Brain Bank for tau Braak stage, Aβ Thal phase, presence of Lewy bodies and p-TDP43. p-TDP43 was assessed according to a condensed protocol to assess limbic-predominant age-related TDP-43 encephalopathy neuropathologic change (LATE-NC), which can detect LATE-NC stages 2–5. All staining for the included cases was negative for p-TDP43 using this method, but we cannot rule out the presence of stage 1 LATE-NC in these samples [40]. Inclusion criteria for AD cases were: clinical dementia diagnosis, Braak stage V–VI and a post-mortem neuropathological diagnosis of AD. Control subjects were chosen to be age/sex matched as much as possible, with inclusion criteria of no diagnosed neurological or psychiatric condition. Where possible, exclusion criteria for both AD and control samples were substantial non-AD neuropathological findings (such as diagnosis of Lewy body dementia (with substantial cortical Lewy bodies in the region of interest), or substantial haemorrhage).

**Table 1:** Demographic and neuropathological characteristics of all human post-mortem subjects

BBN	SD	Clinical diagnosis	Braak stage	Thal phase	Age (years)	Sex	PMI (h)	Lewy body?	Study used
001.35138	SD042/18	Control	0	1	73	F	74	N	Figure 1
001.34150	SD030/18	Control	0	0	63	M	115	N	Figure 1
001.30178	SD024/17	Control	0	0	72	M	60	N	Figure 1
001.35420	SD015/19	Control	I	0	82	F	114	N	Figure 1
001.35215	SD008/19	Control	I	0	82	M	40	N	Figure 1
001.31504	SD046/17	Control	I	0	65	F	76	N	Figure 1
001.31503	SD045/17	Control	I	1	57	F	73	N	Figure 1
001.28402	SD051/15	Control	I	2	79	M	49	N	Figure 1
001.28794	SD018/16	Control	I	0	79	F	72	N	Figure 1
001.30208	SD025/17	Control	I	1	73	M	66	N	Figure 1
001.29528	SD045/16	Control	III	5	93	F	31	N	Figure 1
001.28411	SD006/16	Control	III	2	88	F	65	N	Figure 1
001.26499	SD037/15	Control	III	1	81	F	59	N	Figure 1
001.26491	SD023/15	Control	III	2	77	M	27	Y subcortical	Figure 1
001.20994	SD017/14	Control	III	4	75	M	58	N	Figure 1
001.19600	SD045/13	Control	III	3	85	F	36	N	Figure 1
001.32820	SD008/18	Control	IV	4	69	F	94	N	Figure 1
001.28405	SD052/15	Control	IV	2	84	M	83	N	Figure 1
001.26492	SD013/15	Control	IV	4	74	F	71	N	Figure 1
001.24323	SD050/14	Control	IV	4	67	M	103	N	Figure 1
001.35183	SD005/19	AD	VI	5	74	M	75	Y cortical LBD	Figure 1
001.35096	SD037/18	AD	VI	5	72	M	103	N	Figure 1
001.33698	SD021/18	AD	VI	5	90	F	76	Y brainstem	Figure 1
001.33636	SD017/18	AD	VI	5	93	M	43	Y brainstem	Figure 1
001.32929	SD012/18	AD	VI	5	85	F	80	N	Figure 1
001.30883	SD034/17	AD	VI	5	61	F	69	N	Figure 1
001.29911	SD014/17	AD	VI	5	66	M	52	Y cortical LBD	Figure 1
001.29521	SD035/16	AD	VI	5	95	M	96	N	Figure 1
001.26500	SD039/15	AD	VI	5	81	M	83	N	Figure 1
001.24668	SD058/14	AD	VI	5	96	F	61	N	Figure 1
001.19686	SD063/13	Control	I	1	77	F	75	N	Figure 2
001.26495	SD024/15	Control	I	0	78	M	78	N	Figure 2
001.28406	SD001/16	Control	II	2	79	M	72	N	Figure 2
001.36135	SD012/20	Control	II	1	84	F	30	N	Figure 2
001.28793	SD017/16	Control	II	1	79	F	72	N	Figure 2
001.26718	SD040/15	AD	VI	5	78	M	74	N	Figure 2
001.28771	SD010/16	AD	VI	5	85	M	91	N	Figure 2
001.30973	SD039/17	AD	VI	5	89	F	96	N	Figure 2
001.32929	SD012/18	AD	VI	5	85	F	80	N	Figure 2
001.35182	SD004/19	AD	VI	5	66	M	49	N	Figure 2
001.19686	SD063/13	Control	I	1	77	F	75	N	Figure 3
001.26495	SD024/15	Control	I	0	78	M	78	N	Figure 3
001.29082	SD031/16	Control	III	5	79	F	80	N	Figure 3
001.35181	SD003/19	Control	II	2	82	M	49	N	Figure 3
001.28794	SD018/16	Control	I	0	79	F	72	N	Figure 3
001.24527	SD056/14	AD	V	4	81	M	74	N	Figure 3
001.28771	SD010/16	AD	VI	5	85	M	91	N	Figure 3
001.29695	SD004/17	AD	VI	5	86	M	72	Y limbic	Figure 3
001.30973	SD039/17	AD	VI	5	89	F	96	N	Figure 3
001.28410	SD005/16	AD	V	5	62	F	109	N	Figure 3

*AD* Alzheimer’s disease, *BBN* Medical Research Council Brain Bank Number, *SD* Edinburgh Brain Bank Number, *PMI* postmortem interval

**Fig. 1:**
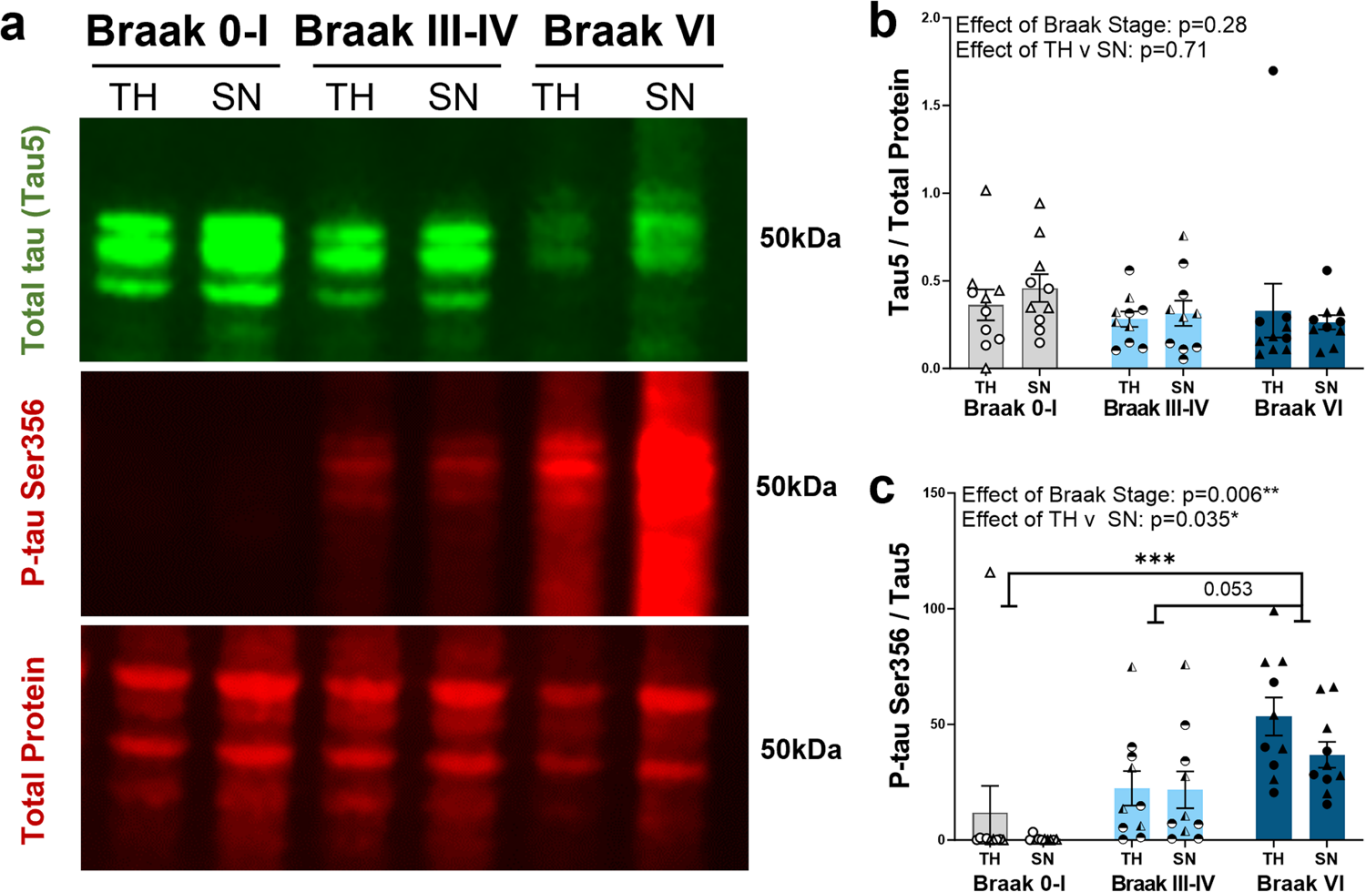
p-tau Ser356 expression increases in a Braak stage-dependent manner in postmortem AD temporal cortex. **a** Representative Western blot image of total homogenates (TH) and synaptoneurosomes (SN) from Braak 0–I, III–IV or VI (diagnosed AD) stage postmortem brain. Blots were probed for total protein (REVERT stain), total tau (Tau-5) and p-tau Ser356. **b** There is no significant impact of Braak stage (*F*_(2,46.01)_ = 1.32, *p* = 0.28) or preparation (TH v SN) (*F*_(1,27.00)_ = 0.15, *p* = 0.71) on the levels of total tau (Tau-5) in human postmortem brain. **c** There is a significant increase in p-tau Ser356 (normalised to total tau) with increasing Braak stage (**F_(2,42.28)_ = 5.72, *p* = 0.006), with a significant difference between Braak 0–I and Braak VI AD (***t_(27)_ = 4.13, *p* = 0.0009) and a strong trend increase between Braak III–IV and Braak VI (*t*_(27)_ = 2.45, *p* = 0.053). There is an effect of preparation (*F_(1,27.00)_ = 4.94, *p* = 0.035), with increased p-tau Ser356 in the total homogenate relative to synaptoneurosomes in Braak VI AD postmortem brains (*t_(27)_ = 2.23, *p* = 0.034). Each point on the graphs represents a single case, triangles = males, circles = females. *N* = 10 cases per Braak stage

**Fig. 2:**
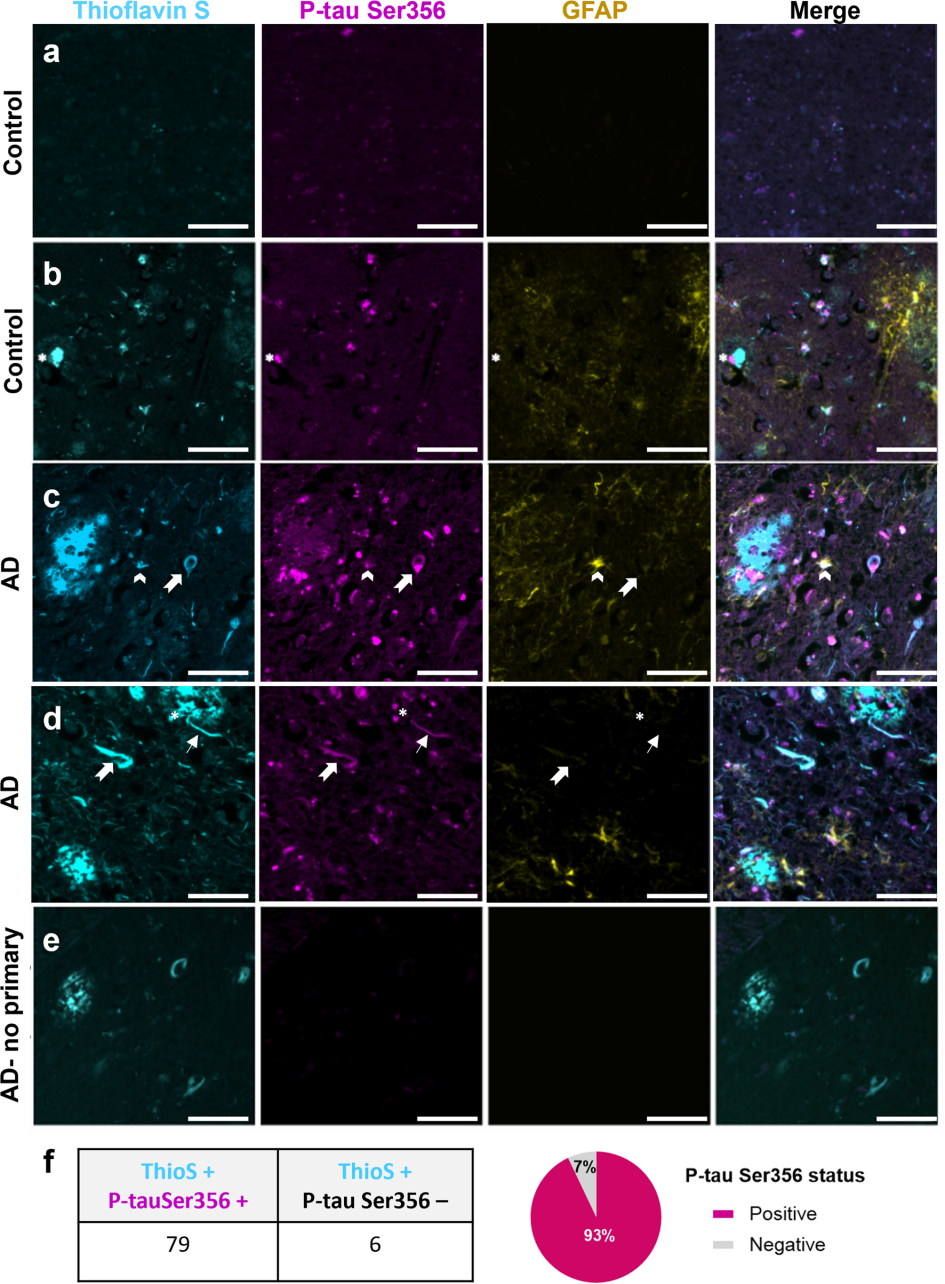
p-tau Ser356 is observed around plaques, in dystrophic neurites, neuropil threads and tangles in human postmortem brain. Paraffin sections of control (**a**, **b**) or AD (**c**–**e**) post-mortem brain stained for plaques/tangles (Thioflavin S—cyan), p-tau Ser356 (magenta) and reactive astrocytes (GFAP—yellow). In human brain sections, p-tau Ser356 (magenta) is found in neurofibrillary tangles (notched arrows), neuropil threads (thin arrows) and dystrophic neurites around plaques (asterisks). p-tau Ser356 is also observed in some GFAP (yellow) positive astrocytes (chevrons). Control brain with little/no Thioflavin S or p-tau Ser356 staining (**a**). Control brain with evidence of dystrophic neurites (**b**), AD brains with evidence of plaques (**c**, **d**), dystrophic neurites (**c**, **d**), neuropil threads (**d**) and tangles (**c**, **d**). No primary antibody control (**e**). Scale bar 50 μm. **f** Quantification of Thioflavin S, p-tau Ser356 double positive tangles, compared to Thioflavin S only tangles shows the vast majority of tangles counted in this study (93%) contain p-tau Ser356

**Fig. 3:**
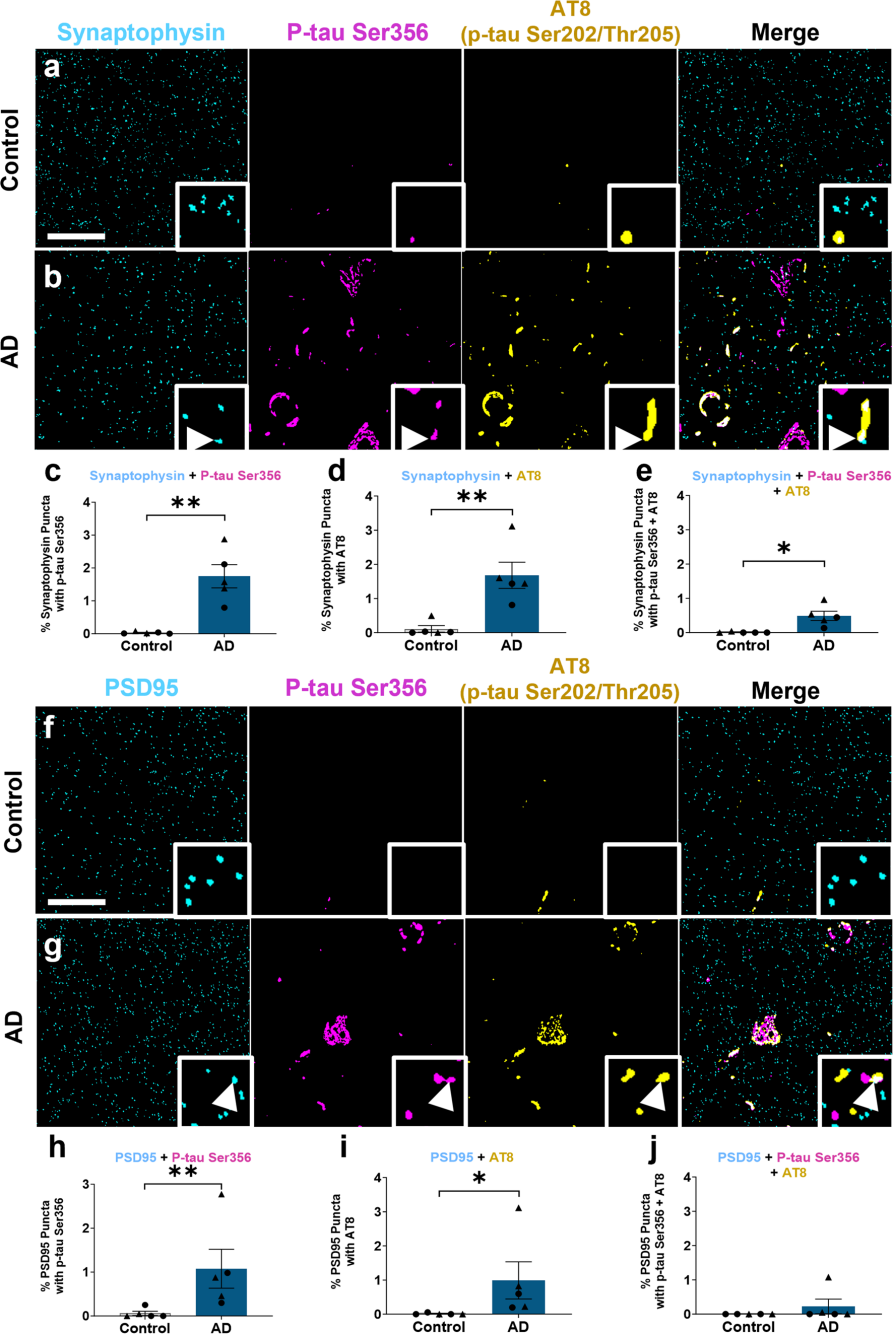
p-tau Ser356 co-localises at the synapse in Alzheimer’s disease post-mortem brain. **a**, **b**, **f**, **g** Representative segmented images of 70 nm thick array tomography sections from control (**a**, **f**) or AD (**b**, **g**) post-mortem brain. Sections were stained with the pre-synaptic marker synaptophysin (cyan **a**, **b**), the postsynaptic marker PSD95 (cyan **f**, **g**), p-tau Ser356 (magenta) and AT8 (yellow). Arrows indicate the area of co-localisation of synaptic markers, p-tau Ser356 and AT8 in AD brain. There is a significant increase in synaptophysin co-localising with p-tau Ser356 (**F_(1,7.14)_ = 18.7, *p* = 0.0033) (**c**), synaptophysin co-localising with AT8 (**F_(1,7.13)_ = 14.6, *p* = 0.0063) (**d**), and synaptophysin containing both p-tau Ser356 and AT8 (*F_(1,7.08)_ = 10.5, *p* = 0.014) (**e**) in AD brain. There is a significant increase in PSD-95 co-localising with p-tau Ser356 (**F_(1,7.38)_ = 12.50, *p* = 0.0088) (**h**) and PSD-95 co-localising with AT8 in AD brain (**F_(1,7.14)_ = 9.08, *p* = 0.019) (**i**), but no significant difference in post-synapses containing both p-tau Ser356 and AT8 (*F*_(1,7.27)_ = 1.28, *p* = 0.294) between control and AD (**j**). Each point on the graph represents the median from a single case, males = triangles, females = circles. *N* = 5 control and 5 AD cases. Scale bar represents 20 µm, square insets represent 5 µm × 5 µm

### Human postmortem total protein homogenate and synaptoneurosome preparation

At the MRC Edinburgh Brain Bank, fresh postmortem brain tissue was sectioned into small blocks of around 500 mg each, flash frozen in on dry ice then stored at − 80 °C until requested by the research group. Ten different frozen postmortem human brain case samples were obtained per Braak stage for the Brodmann area 20/21 region (summary details listed in Table 2**)**. Total protein homogenate and synaptoneurosomes were generated as previously described [29]. For each case sample, 300–500 mg of frozen tissue was thawed and immediately homogenised, on ice, in a glass-teflon dounce homogeniser in 1 ml of homogenisation buffer (25 mM HEPES (pH 7.5), 120 mM NaCl, 5 mM KCl, 1 mM MgCl_2_, 2 mM CaCl_2_, protease and phosphatase inhibitors (Roche complete mini: 11836153001)). The homogenate was then flushed through an 80 μm-pore nylon filter (Millipore NY8002500), with 300 μl of the resulting crude total homogenate set aside and frozen at − 80 °C. To generate synaptoneurosome preps, total homogenate was further filtered through a 5 μm filter (Millipore SLSV025NB). Synaptoneurosomes were centrifuged at 1000xg for 5 min and the pellet was collected. Proteins (either total homogenate or synaptoneurosome) were extracted in an SDS buffer (100 mM Tris–HCl, 4% SDS) and the protein concentration determined by commercial BCA assay before equal protein amounts were diluted in 2 × Laemmli buffer, boiled then loaded for Western blot (below).

**Table 2:** Summary demographic information of human postmortem subjects (homogenate Western blot study (Fig. 1 and Supp. Fig. 1))

Braak stage	0–I (*N* = 10)	III–IV (*N* = 10)	VI (*N* = 10)	Overall (*N* = 30)
Age (years)				
Mean (SD)	72.5 (8.51)	79.3 (8.37)	81.3 (12.5)	77.7 (10.4)
Median [Min, Max]	73.0 [57.0, 82.0]	79.0 [67.0, 93.0]	83.0 [61.0, 96.0]	78.0 [57.0, 96.0]
Sex				
F	5 (50.0%)	6 (60.0%)	4 (40.0%)	15 (50.0%)
M	5 (50.0%)	4 (40.0%)	6 (60.0%)	15 (50.0%)

### Human postmortem immunohistochemistry

(Summary patient details in Table 3) 4 μm sections of formalin-fixed, paraffin-embedded tissue from Broadmann area 20/21 (inferior temporal gyrus) were de-waxed in xylene, then decreasing concentrations of ethanol, incubated with autofluorescence inhibitor reagent (Millipore 2160), and non-specific antigens blocked by incubation in a solution of 0.1 M PBS containing 0.3% Triton X-100 and 10% normal donkey serum for 1 h. Primary antibodies to p-tau Ser356 (Abcam: ab75603, diluted 1:1000) and GFAP (Abcam: ab4674, diluted 1:3000) were diluted in blocking buffer and incubated on sections overnight at 4 °C. Sections were washed with 0.1 M PBS containing 0.3% triton X-100, and then incubated in secondary antibodies (donkey anti-rabbit Alexa-fluor 594, Invitrogen A21207 and donkey anti-chicken Alexa-fluor 647, Invitrogen A78952) diluted 1:200 in block buffer at room temperature for 1 h. Sections were washed with PBS, then incubated in 0.5% Thioflavin S in 50% ethanol, 50% H_2_O solution for 8 min in the dark at room temperature. Slides were dipped in 80% ethanol to reduce Thioflavin S background, and then rinsed thoroughly in distilled water before coverslipping with Immu-Mount™ (Epredia™: 9990402). Sections were imaged with a 20 × 0.8NA objective on a Zeiss AxioImager Z2 microscope equipped with a CoolSnap camera. Ten regions of interest were imaged in the cortex of each slide sampling in a systematic random fashion to sample throughout all six cortical layers. Images were examined in Fiji (ImageJ) by a blinded experimenter. Neurofibrillary tangles were identified by Thioflavin S staining in a flame shape with a hole in the centre (where the nucleus resides) at the approximate size of a neuronal cell body (~ 10 μm in diameter). Cells with Thioflavin S and GFAP staining were excluded to exclude astrocytic tau pathology. Dystrophic neurites were identified as p-tau Ser356 containing swellings of > 5 μm in diameter associated with Thioflavin-S-positive Aβ plaques. Neuropil threads were identified as Thioflavin S and/or p-tau Ser356 staining in linear thread-like patterns consistent with axonal or dendritic staining. The presence or absence of p-tau Ser356 was noted for each identified neurofibrillary tangle and quantified as a percentage of total detected tangles in the ten regions of interest for each case.

**Table 3:** Summary demographic information of human postmortem subjects (paraffin sections study (Fig. 2))

	Control (*N* = 5)	AD (*N* = 5)	Overall (*N* = 10)
Age (years)			
Mean (SD)	79.4 (2.70)	80.6 (9.07)	80.0 (6.34)
Median [Min, Max]	79.0 [77.0, 84.0]	85.0 [66.0, 89.0]	79.0 [66.0, 89.0]
Sex			
F	3 (60.0%)	2 (40.0%)	5 (50.0%)
M	2 (40.0%)	3 (60.0%)	5 (50.0%)

### Human postmortem array tomography

Tissue from non-AD control patients, with Braak stages I–II and Thal phase 1–2, and AD patients with Braak stage VI and Thal phase 5 were acquired (summary patient details in Table 4 and Supplementary Table 2). As described previously [9], fresh tissue was fixed in 4% paraformaldehyde for 3 h, dehydrated in ethanol and embedded in LR White Resin. A diamond knife (Diatome) mounted onto an Ultracut microtome (Leica) was used to cut the embedded tissue into 70 nm serial sections. For Fig. 3, 15–30 serial section ribbons were collected onto gelatin-coated coverslips and immunostained with the following primary antibodies for 1 h: 1:500 Rabbit p-tau Ser356 (Abcam: ab75603), 1:100 goat synaptophysin (R&D Systems: AF555), 1:200 chicken GFAP (Abcam: ab4674), 1:200 guinea pig PSD95 (Synaptic Systems: 124 014) and 1:100 mouse p-tau Ser202/Thr205 (AT8) (Thermo Fisher: MN1020). Secondary antibodies, donkey anti-rabbit Alexa-fluor 405 (Abcam: ab175651), donkey anti-goat Alexa-fluor 594 (Abcam: ab150136), goat anti-chicken Alexa-fluor 405 (Invitrogen: A48260), goat anti-guinea pig Alexa-fluor 488 (Abcam: ab150185) and donkey anti-mouse Alexa-fluor 647 (Thermo Fisher: A32787) were then applied at 1:50 concentration to ribbons for 45 min. Images were obtained with a 63 × 1.4 NA objective on a Leica TCS confocal microscope. Details of staining and imaging for Supp. Fig. 2 can be found in supplemental information. Images from the same locus in each serial section along a ribbon were then aligned, thresholded, and parameters quantified using in-house scripts in Fiji (ImageJ) and MATLAB. Any signals that do not appear in the same x–y location in a least two adjacent (z) brain sections are discarded as noise. Resulting parameter data were statistically analysed using custom R Studio scripts. Analysis code is available on GitHub (https://github.com/Spires-Jones-Lab).

**Table 4:** Summary demographic information of human postmortem subjects (array tomography study (Fig. 3))

	Control (*N* = 5)	AD (*N* = 5)	Overall (*N* = 10)
Age (years)			
Mean (SD)	79.0 (1.87)	80.6 (10.8)	79.8 (7.35)
Median [Min, Max]	79.0 [77.0, 82.0]	85.0 [62.0, 89.0]	80.0 [62.0, 89.0]
Sex			
F	3 (60.0%)	2 (40.0%)	5 (50.0%)
M	2 (40.0%)	3 (60.0%)	5 (50.0%)

### Animals

APP/PS1 (APPswe, PSEN1dE9 [31]) and wildtype litter mate male and female mouse pups, aged 6–9 days old (P6-9), were obtained from a breeding colony at the Bioresearch and Veterinary Services Animal Facility at the University of Edinburgh. Animals were culled via cervical dislocation, performed by a trained individual, who was assessed by the Named Training and Competency Officer (NTCO). All animal work was conducted according to the Animals (Scientific Procedures) Act 1986 under the project licence PCB113BFD and PP8710936. All animals were bred and maintained under standard housing conditions with a 12/12 h light–dark cycle.

### Mouse organotypic brain slice culture generation and maintenance

Mouse organotypic brain slice cultures (MOBSCs) were generated and maintained as described previously [19, 20, 28, 56], with minor modifications. Mouse pups aged postnatal day (P)6–9 were culled by cervical dislocation. Brains were rapidly transferred to ice-cold 0.22 μm-filtered dissection medium composed of 87 mM NaCl, 2.5 mM KCl, 25 mM NaHCO_3_, 1.25 mM NaH_2_PO_4_, 25 mM glucose, 75 mM sucrose, 7 mM MgCl_2_, 0.5 mM CaCl_2_, 1 mM Na-Pyruvate, 1 mM Na-Ascorbate, 1 mM kynurenic acid and 1× penicillin/streptomycin (Thermo Fisher: 15140122) (340 mOsm, pH 7.4), bubbled with 95% O_2_, 5% CO_2_. All salts and chemicals were purchased from Merck. Brains were then mounted on and glued (cyanoacrylate, Loctite) to a vibratome stage. A Leica VT1200S vibratome was used to cut 350 µm-thick horizontal slices, from which the hippocampus was dissected with fine needles. Hippocampal slices were plated on membranes (Millipore: PICM0RG50) sitting on top of 1 ml of maintenance medium, placed inside 35 mm culture dishes. The maintenance medium was 0.22 µm-filtered and composed of MEM with Glutamax-1 (50%) (Invitrogen: 42360032), heat-inactivated horse serum (25%) (Thermo Fisher: 26050070), EBSS (18%) (Thermo Fisher: 24010043), d-glucose (5%) (Sigma: G8270), 1× penicillin/streptomycin (Thermo Fisher: 15140122), nystatin (3 units/ml) (Merck: N1638) and ascorbic acid (500 μM) (Sigma-Aldrich: A4034). Slice cultures were then immediately placed into an incubator and maintained at 37 °C with 5% CO_2_ thereafter. The slice culture medium was changed fully within 24 h of plating, then at 4 days in vitro (div), 7 div and weekly thereafter. Four culture dishes were made per pup, with 1–2 slices plated per dish. Cultures were kept for either 2 weeks or 4 weeks, with 5 μM WZ4003 (APExBIO: B1374) or DMSO (Sigma-Aldrich: D2438) control applied in culture medium at every feed during the treatment period (either 0–2 weeks or 2–4 weeks).

### Human brain slice cultures

Human brain slice cultures (HBSCs) were generated from surplus neocortical access tissue from patients undergoing tumour resection surgery, with ethical approval from the Lothian NRS Bioresource (REC number: 15/ES/0094, IRAS number: 165488) under approval number SR1319. Additional approval was obtained for receiving data on patient sex, age, reason for surgery and brain region provided (NHS Lothian Caldicott Guardian Approval Number: CRD19080). Patient details are listed in Table 5. The informed consent of patients was obtained using the Lothian NRS Bioresource Consent Form. Dissection and culture methods have been adapted from published studies [41, 51, 53, 54]. Access, non-tumour, neocortical tissue was excised from patients (would normally be disposed of during surgery) and immediately placed in sterile ice-cold oxygenated 0.22 μm-filtered artificial cerebrospinal fluid (aCSF) containing 87 mM NaCl, 2.5 mM KCl, 10 mM HEPES, 1.62 mM NaH_2_PO_4_, 25 mM d-glucose, 129.3 mM sucrose, 1 mM Na-Pyruvate, 1 mM ascorbic acid, 7 mM MgCl_2_, and 0.5 mM CaCl_2_. The tissue was then sub-dissected and mounted in 2% agar, before being glued (cyanoacrylate, Loctite) to a vibratome stage. 300 µm-thick slices were then cut in ice-cold and oxygenated aCSF, before being sub-dissected into smaller slices. Slices were placed into 0.22 μm-filtered wash buffer composed of oxygenated Hanks Balanced Salt Solution (HBSS, Thermo Fisher: 14025092), HEPES (20 mM) and 1X penicillin–streptomycin (Thermo Fisher: 15140122) (305 mOsm, pH 7.3) for 15 min at room temperature. Slices were then plated on membranes (Millipore: PICM0RG50) sitting on top of 750 μl of a second wash medium, placed inside 35 mm culture dishes. The second wash medium was 0.22 µm-filtered and composed of BrainPhys Neuronal Medium (StemCell Technologies: 5790) (96%), N2 (Thermo Fisher: 17502001) (1×), B27 (Thermo Fisher: 17504044) (1×), hBDNF (StemCell Technologies: 78005) (40 ng/ml), hGDNF (StemCell Technologies: 78058) (30 ng/ml), Wnt7a (Abcam: ab116171) (30 ng/ml), ascorbic acid (2 μM), dibutyryl cAMP (APExBIO: B9001) (1 mM), laminin (APExBIO: A1023) (1 ug/ml), penicillin/streptomycin (Thermo Fisher: 15140122) (1×), nystatin (Merck: N1638) (3 units/ml) and HEPES (20 mM). Slice cultures were kept in the second wash medium in an incubator at 37 °C with 5% CO_2_ for 1 h, after which the medium was aspirated and replaced with maintenance medium. The maintenance medium composition was identical to that of the second wash medium, but without HEPES. For all human cases, the time taken from patient tissue removal to the brain slices being placed in the incubator (post-operation interval) was kept under 2 h. 100% medium exchanges occurred twice weekly thereafter. Cultures were kept for 2 weeks and treated with either 10 μM WZ4003 (APExBIO: B1374) or DMSO (Sigma-Aldrich: D2438) control applied in culture medium, and with every feed thereafter from 0 days in vitro (div). To assess neuronal integrity in HBSCs, 14 div slices were fixed overnight in 4% PFA, washed 3 × in PBS, blocked for 1 h in PBS + 3% normal goat serum + 0.5% Triton X-100 and then incubated overnight at 4 °C in 1:500 guinea pig anti-MAP2 (Synaptic systems: 188004) in blocking solution. The slices were then washed 3 × in PBS, before incubation for 2 h in 1:500 secondary antibody (goat anti-guinea pig-488 (Thermo Fisher: A11073)) in block buffer at room temperature. Slices were washed 3 × in PBS, counterstained with DAPI for 15 min, washed 3 × in PBS then mounted on slides in Vectashield Antifade mounting medium (2B Scientific: H-1900). Images were taken using a 63 × 1.4 NA objective on a Leica TCS confocal microscope.

**Table 5:** Demographic, brain region and reason for surgery information for human samples obtained from neurosurgical procedures

Brain region	Reason for surgery	Age (years)	Sex
Right frontal	Glioblastoma	37	F
Left temporal	Glioma	65	M
Right temporal	Metastatic brain tumour	76	F
Left frontal	Glioma	54	M
Left frontal	Metastatic brain tumour	52	F

Tissue obtained from these individuals went on to generate the HBSCs used in Fig. 6

### Western blots

MOBSCs or HBSCs were removed from the culture membrane with a scalpel blade into RIPA buffer (Thermo Fisher Scientific: 89901) with protease inhibitor cocktail (1×) and EDTA (1×) (Thermo Fisher Scientific: 78429). Slices were thoroughly homogenised via trituration through 20 pipette fill and empty cycles (using a 100 μl pipette tip). 50 μl of RIPA buffer was used per MOBSC slice and 100 μl of RIPA per HBSC slice. RIPA-buffered MOBSCs, HBSC samples, or human postmortem total protein/synaptoneurosome samples were then mixed into equal volumes of 2X Laemmli buffer (Merck: S3401-10VL) and boiled for 10 min at 98 °C. 12 µl of each sample was loaded into 4–12% NuPage Bis–Tris gels (Invitrogen: NP0336BOX), before proteins were separated by electrophoresis using MES SDS running buffer (Invitrogen: NP0002). Proteins were then transferred onto PVDF transfer membranes (Invitrogen: IB24002), before a total protein stain (Li-Cor Biosciences: 926-11016) image was acquired using a Li-Cor Odyssey Fc machine. Membranes were de-stained and then blocked for 1 h using PBS Intercept Blocking Buffer (Li-Cor Biosciences: 927-70001). Primary antibodies were diluted in PBS Intercept Blocking Buffer with 0.1% Tween-20 and incubated with membranes overnight at room temperature, with shaking. Membranes were washed three times for 5 min with PBS-Tween, then incubated in darkness for 2 h with IRDye anti-rabbit (Li-Cor Biosciences: 680RD) and anti-mouse (Li-Cor Biosciences: 800CW) secondary antibodies, each at 1:10,000 concentration. Membranes were washed 3 × in PBS-Tween, 1 × in PBS and then imaged using a Li-Cor Odyssey Fc machine. The following primary antibodies were used: 1:500 mouse Tau-5 (Abcam: AB80597), 1:1000 rabbit ps356 tau (Abcam: AB75603), 1:500 rabbit PSD-95 (Abcam: AB18258), 1:2500 rabbit Tuj-1 (Sigma: T2200), 1:500 rabbit p-tau Ser202/Thr205 (Cell Signalling Technology: 30505S), 1:500 rabbit NUAK1 (ProteinTech 22723-1-AP) and 1:2000 rabbit cyclophilin-B (Abcam: AB16045). Western blot images were analysed using Empiria Studio (Version 2.3).

### Statistics

**Fig. 4:**
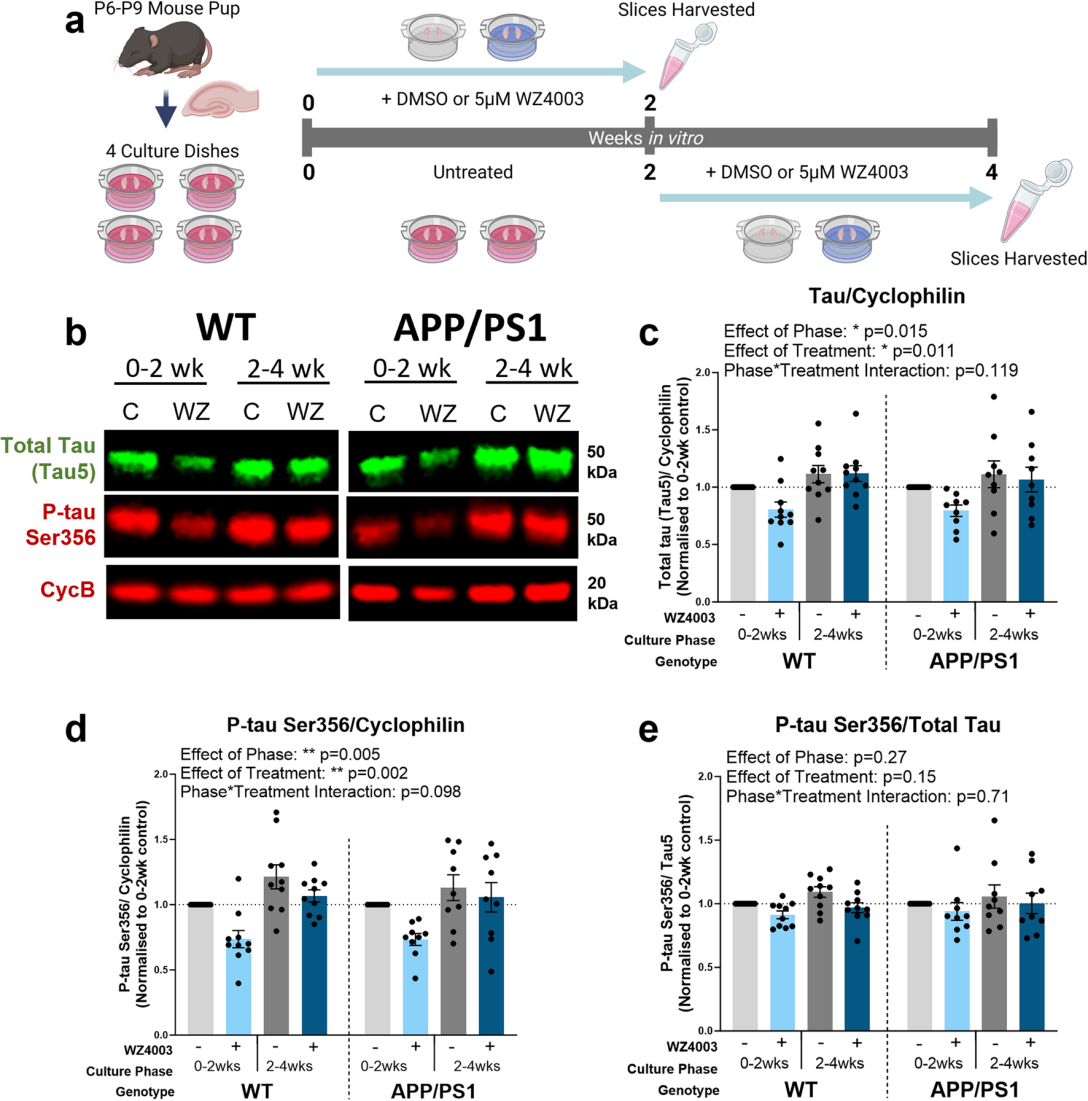
WZ4003 lowers total tau and p-tau Ser356 in a phase-dependent manner in MOBSCs. **a** Schematic showing the dissection and treatment schedule for MOBSCs. Four dishes are generated per animal, split into four treatment conditions: 0–2 weeks control, 0–2 weeks WZ4003, 2–4 weeks control, 2–4 weeks WZ4003. Slices are harvested for Western blot at the end of their treatment phase and ratio change in protein levels (normalised to cyclophilin-B) compared within samples from the same mouse. **b** Representative Western blot for total tau, p-tau Ser356 and housekeeping protein cyclophilin. The following graphs are displayed normalised to 0–2 week control for each animal to show relative differences, but statistics are performed on absolute data (absolute data displayed in Supp Fig. 3). **c** There is a significant effect of phase (*F_(1,51.00)_ = 6.28, *p* = 0.015) and treatment (*F_(1,51.00)_ = 7.01, *p* = 0.011) on the levels of total tau, but no effect of genotype (*F*_(1,21.30)_ = 0.02, *p* = 0.894). **d** There is a significant effect of phase (**F_(1,51.00)_ = 8.73, *p* = 0.005) and treatment (**F_(1,51.00)_ = 10.7, *p* = 0.002) on the levels of p-tau Ser356, and a trend interaction between Phase*Treatment (*F*_(1,51.00)_ = 2.83, *p* = 0.098), but there is no effect of genotype (*F*_(1,26.41)_ = 0.00, *p* = 0.997). **e** There are no significant effects of phase (*F*_(1,51.00)_ = 1.25, *p* = 0.27), treatment (*F*_(1,51.00)_ = 2.10, *p* = 0.15) or genotype (*F*_(1,26.51)_ = 0.08, *p* = 0.781) on the p-tau Ser356/total tau ratio. *N* = 9 APP/PS1 and 10 WT animals, 1–2 slices per animal per condition. Cartoons generated using BioRender

**Fig. 5:**
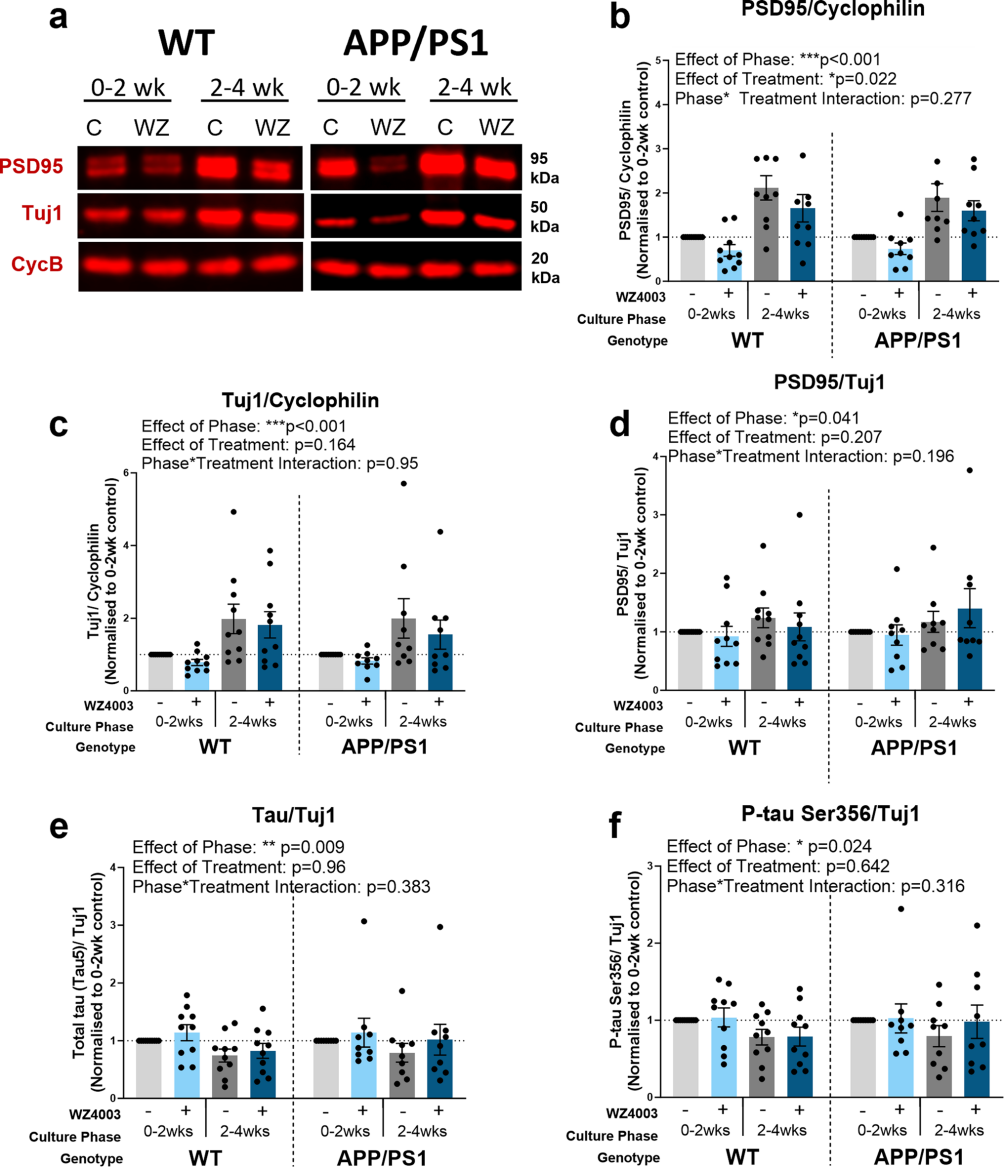
Neuronal and synaptic proteins increase over time in culture and are impacted by WZ4003 treatment. **a** Representative Western blot for PSD95, Tuj1 and housekeeping protein cyclophilin. The following graphs are displayed normalised to 0–2 week control for each animal to show relative differences, but statistics are performed on absolute data (absolute data displayed in Supp Fig. 3). **b** There is a significant effect of phase (***F_(1,51.00)_ = 26.7, *p* < 0.001) and treatment (*F_(1,51.00)_ = 5.60, *p* = 0.022) on the levels of PSD95 normalised to cyclophilin, but no effect of genotype (*F*_(1,41.92)_ = 0.21, *p* = 0.652). **c** There is a significant effect of phase (***F_(1,51.00)_ = 13.4, *p* < 0.001) on the levels of Tuj1 normalised to cyclophilin, but no effects of treatment (*F*_(1,51.00)_ = 1.99, *p* = 0.164) or genotype (*F*_(1,41.92)_ = 0.00, *p* = 0.957). **d** There is a significant effect of phase (*F_(1,51.00)_ = 4.39, *p* = 0.041) but no effect of treatment (*F*_(1,51.00)_ = 1.63, *p* = 0.207) or genotype (*F*_(1,47.89)_ = 0.13, *p* = 0.715) on the levels of PSD95 when normalised to Tuj1. **e** There is a significant effect of phase (**F_(1,51.00)_ = 7.38, *p* = 0.009), but no effect of treatment (*F*_(1,51.00)_ = 0.00, *p* = 0.964) or genotype (*F*_(1,42.38)_ = 0.08, *p* = 0.774), in the levels of total tau when normalised to Tuj1. **f** There is a significant effect of phase (*F_(1,51.00)_ = 5.40, *p* = 0.024), but no effect of treatment (*F*_(1,51.00)_ = 0.22, *p* = 0.642) or genotype (*F*_(1,42.83)_ = 0.00, *p* = 0.957), on the levels of p-tau Ser356 when normalised to Tuj1. *N* = 9 APP/PS1 and 10 WT animals, 1–2 slices per animal per condition

**Fig. 6:**
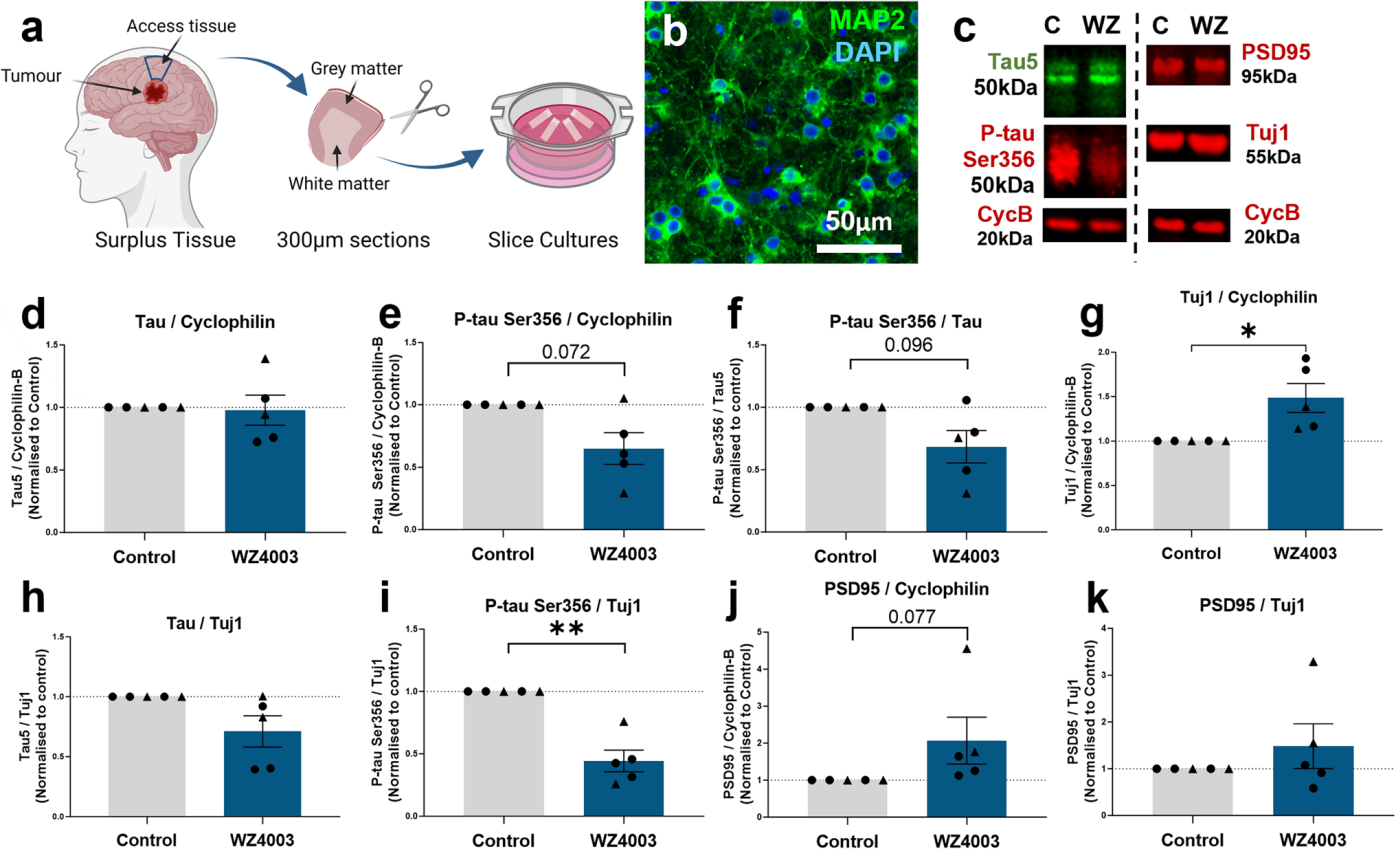
Human slice cultures are responsive to WZ4003 treatment. **a** Cartoon illustrating the work flow for generating human brain slice cultures (HBSCs) from surplus access tissue from neurosurgical procedures. **b** MAP2 (green) and DAPI (blue) staining shows intact neuronal cell bodies and neurite processes in 14 div HBSC (scale bar = 50 μm). **c** Representative Western blot from 14 div HBSC showing total tau (tau5), p-tau Ser356, PSD95, Tuj1 and housekeeping protein cyclophilin-B. **d** WZ4003 does not significantly alter levels of tau (normalised to cyclophilin) (*t*_(4)_ = 0.43, *p* = 0.688). **e** There is a trend for WZ4003 treatment to reduce p-tau Ser356 (normalised to cyclophilin) (*t*_(4)_ = 2.43, *p* = 0.072). **f** There is a trend for WZ4003 to reduce the ratio of p-tau Ser356/total tau (*t*_(4)_ = 2.17, *p* = 0.096). **g** WZ4003 treatment significantly increased Tuj1 levels (*t_(4)_ = 3.39, *p* = 0.028). **h** WZ4003 does not significantly alter tau levels as a proportion of neuronal protein (*t*_(4)_ = 2.04, *p* = 0.11). **i** WZ4003 significantly lowers p-tau Ser356 as a proportion of neuronal protein (**t_(4)_ = 4.81, *p* = 0.0086). **j** There is a trend for WZ4003 to increase PSD95 protein (normalised to cyclophilin) (*t*_(4)_ = 2.37, *p* = 0.077). **k** There is no effect of WZ4003 on PSD95 protein (normalised to Tuj1) (*t*_(4)_ = 0.74, *p* = 0.501). *N* = 5 cases per condition. Each point represents an individual human case, triangles = males, circles = females. Cartoons generated using BioRender

All data were analysed using R (v 4.2.2) and R Studio (v 2023.03.1, Build 446). Statistical tests were chosen according to the experimental design and dataset type. Unpaired *T* tests, ratio paired *T *tests and N-way repeated-measures ANOVA tests (*car* package) were conducted using linear mixed effects models (LMEM) using the *lme4* package (each model is listed in the relevant results section). Human or mouse case was included as a random effect in linear mixed effects models to avoid pseudoreplication of the data [57]. *T* tests used Satterthwaite’s method, whilst Type-III F-Wald ANOVA tests used the Kenward–Roger method, to compute degrees of freedom. For N-way ANOVA analyses resulting in significant interaction effects or significant main effects where more than two within-factor levels existed, multiple comparison tests using the Tukey method for adjusting *p* values were computed with the *emmeans* package. Model assumptions were tested using Shapiro–Wilk and *F* tests, along with plotting model residuals. To assess equality of variance, standardised model residual scatter was plotted against fitted values. Residual distribution was assessed for normality by plotting a histogram and kernel density estimate of standardised model residuals over a standard normal distribution, and by generating a quantile–quantile plot of dataset quantiles against normal quantiles. Datasets in Fig. 3 were Tukey-transformed prior to performing statistical tests if assumptions of normality were violated. Statistical tests performed on datasets represented in Figs. 4, 5, 6 were conducted on absolute data that was log-transformed, to compute ratio differences between differentially treated samples from the same animal/human and to satisfy assumptions of normality. Figures 4, 5 and 6 represent control-normalised data to display within-animal and within-human case effects. For the Thal phase correlation graph data represented in Supp. Fig. 1, a Spearman’s rank correlation test was performed for each preparation group (TH or SN) due to data being non-normally distributed. Significance values are reported as *p* < 0.05 *, *p* < 0.01 *, *p* < 0.001 *** and error bars represent the mean ± SEM.

## Results

### p-tau Ser356 increases in a Braak stage-dependent manner in human postmortem temporal cortex

To investigate how the levels of p-tau Ser356 change with Braak stage in postmortem human temporal cortex (BA20/21), soluble fractions of total homogenate and synaptoneurosome preparations were generated from frozen postmortem brain from ten Braak 0–I individuals, ten Braak III–IV individuals and ten Braak VI individuals (with clinically diagnosed AD). Individual patient details are listed in Table 1 with summary demographic information listed in Table 2. Levels of p-tau Ser356 and total tau (tau5) were quantified by Western blot (Fig. 1a). Total tau (tau5) was detected in all cases and was normalised to total protein (REVERT protein stain). For statistical analysis, the following LMEM was applied: *Protein level* ~ Braak Stage * Preparation + *(1|CaseID).* The levels of total tau did not increase with Braak stage (Fig. 1b, effects of Braak stage: *F*_(2,46.01)_ = 1.32, *p* = 0.28) and there were no detectable differences in the levels of tau in total homogenate compared to synaptoneurosome preparations (Fig. 1b, effect of preparation: *F*_(1,27.00)_ = 0.15, *p* = 0.71). By contrast, the levels of p-tau Ser356, normalised to total tau levels, showed a significant increase with Braak stage (Fig. 1c effect of Braak stage: **F_(2,42.28)_ = 5.72, *p* = 0.006) and a detectable effect of preparation (Fig. 1c, effect of preparation: *F_(1,27.00)_ = 4.94, *p* = 0.035) driven by an increase in p-tau Ser356 in total homogenate, compared to synaptoneurosome, in Braak VI cases (Fig. 1c, multiple comparisons test: *p = 0.034). This is possibly reflective of increased tau in the cell bodies at a stage where tangle prevalence will be high. There was a significant increase in p-tau Ser356 in Braak VI AD brains compared to Braak 0–I control brains (Fig. 1c, multiple comparisons test: ***p = 0.0009), and a strong trend for increase between Braak III–IV and Braak VI (Fig. 1c, multiple comparisons test: *p* = 0.053). A similar pattern was seen when examining the levels of p-tau Ser202/Thr205 (a well characterised phosphoepitope known to be upregulated in later Braak stages [45]) (Supp. Fig. 1a,c), with a significant increase in the ratio of p-tau Ser202/Thr205/total tau with Braak stage (Supp. Fig. 1c, effect of Braak stage: ***F_(2,32.12)_ = 10.7, *p* < 0.001). Interestingly, in contrast to previous studies which found increased levels of NUAK1 in AD versus control samples[37], we did not see an overall effect of Braak stage on soluble NUAK1 levels (Supp. Fig. 1b,d) (effect of Braak stage: *F*_(2,36.83)_ = 0.64, *p* = 0.531). We did, however, find an accumulation of NUAK1 in the synaptoneurosome compartment, relative to the total homogenate in Braak stages III–IV or VI brains (but not in Braak 0–I brains), indicating that there may be a shift in location of NUAK1 throughout disease progression (Supp. Fig. 1d, effect of Braak stage*preparation: *F_(2,27.00)_ = 3.48, *p* = 0.045). When analysing correlation of protein levels with Aβ Thal phase, we found a significant correlation between increasing Thal phase and increased levels of both p-tau Ser356 (Supp. Fig. 1e) and p-tau Ser202/Thr205 (Supp. Fig. 1f). For NUAK1, we did not see a significant correlation with Thal phase, but there appeared to be divergence between synaptoneurosome and total homogenates in the later stages (Supp. Fig. 1 g).

### Co-localisation of p-tau Ser356 with Thioflavin-S-positive tangles, dystrophic neurites and neuropil threads in AD postmortem brain tissue

Having established a significant increase in p-tau Ser356 in AD brains, we sought to establish whether this phosphoepitope of tau is found in pathological lesions including neurofibrillary tangles (NFTs), dystrophic neurites around plaques and reactive astrocytes. Paraffin sections were obtained from five control (Braak 0–II, Thal 0–2) and five confirmed AD (Braak VI, Thal 5) cases (individual patient details listed in Table 1, summary demographic details listed in Table 3). Staining in paraffin sections demonstrated that p-tau Ser356 is readily detectable in tangles, dystrophic neurites, neuropil threads and some reactive astrocytes in AD brain (Fig. 2). Of the 85 Thioflavin-S-positive tangles, we identified in the AD cases, 79 were positive for p-tau Ser356 (93%) (Fig. 2f). Interestingly, whilst control brains showed, as expected, significantly lower levels of neuropathology (Fig. 2a), we also found evidence of p-tau Ser356 staining in some control brains, in areas co-localising with Thioflavin S, most notably in dystrophic neurites (Fig. 2b). Together, these results suggest that p-tau Ser356 is a common component of dystrophic neurites and Thioflavin-S-positive NFTs, and may, therefore, be involved early in the tau aggregation cascade.

### p-tau Ser356 co-localises with pre- and post-synaptic terminals in AD postmortem brain tissue

Given recent work highlighting the role of synaptic tau in the early stages of AD pathology [9], we next sought to examine the presence of p-tau Ser356 at synaptic terminals. Here we used array tomography, which physically overcomes the diffraction limit of light in the axial plane to enable imaging of the protein composition of individual synapses [33]. Effective detection of proteins located within synapses using this method has been validated against super-resolution and electron microscopy imaging [9, 33, 48, 52]. We imaged five confirmed AD (Braak V–VI) and five age/sex-matched control cases (Braak 0–III) for p-tau Ser356, AT8 (tau phosphorylated at Ser202 and Thr205) and the pre-synaptic marker synaptophysin (Fig. 3a–e) or post-synaptic marker PSD95 (Fig. 3f–j). Individual patient demographic data are listed in Table 1 and summary demographic data are listed in Table 4. For statistical analysis, the following LMEM was applied: *Co-localisation %* ~ *Diagnosis* + *Sex* + *(1|CaseID)*. Whilst largely undetectable in control cases (Fig. 3a), both p-tau Ser356 (Fig. 3c) and AT8 (Fig. 3d) were found to co-localise with synaptophysin in AD cases (**F_(1,7.14)_ = 18.7, *p* = 0.0033 and **F_(1,7.13)_ = 14.6, *p* = 0.0063, respectively). Both p-tau Ser356 (Fig. 3h) and AT8 (Fig. 3i) were also found to co-localise with PSD95 in AD brains (**F_(1,7.38)_ = 12.5, *p* = 0.0088, *F_(1,7.14)_ = 9.08, *p* = 0.019, respectively). To provide additional confirmation that p-tau Ser356 is located *within* synaptic compartments, we conducted Förster resonance energy transfer (FRET) imaging of AD brain samples (see Supplementary methods) [69]. We find that p-tau Ser356 is located within 10 nm of both synaptophysin (Supp. Fig. 2c: *t_(4)_ = 4.18, *p* = 0.014) and PSD95 (Supp. Fig. 2f: ***t_(5)_ = 7.56, *p* = 0.0006).

Interestingly, whilst p-tau Ser356 and AT8 were found to co-localise with a similar percentage of pre-synaptic terminals (median of 1.38% (Fig. 3c) and 1.60% (Fig. 3d), respectively), the percentage of synaptophysin puncta that co-localised with both tau epitopes, (whilst still significantly higher in AD than controls (*F_(1,7.08)_ = 10.5, *p* = 0.014)), was considerably smaller (median of 0.36% (Fig. 3e)). For post-synapses, there was no significant increase in the number of puncta that co-localised with both epitopes in AD compared to control brain (Fig. 3j F_(1,7.27)_ = 1.28, *p* = 0.294). This could suggest that different synapses have unique signatures of tau phosphorylation patterns in AD, or that there are technical considerations (e.g. masked antibody binding sites) that make it harder to resolve both epitopes when they co-localise.

In light of recent findings that astrocytes ingest synapses in post-mortem AD brain, we examined the levels of pre- (Supp. Fig. 2a,b) and post-synapses (Supp. Fig. 2d,e) contained *within* astrocytes [59, 62], and whether synapses containing p-tau Ser356 were more likely to be ingested. We found that pre-synapses containing p-tau Ser356 were around 5 × more likely to be found within astrocytes (Supp. Fig. 2b: **F_(1,14)_ = 9.94, *p* = 0.00705), than the general pre-synapse population, whilst there was no effect on post-synapse ingestion with tau status (Supp. Fig. 2e: *F*_(1,17)_ = 0.0341, *p* = 0.856).

### The NUAK inhibitor WZ4003 lowers p-tau Ser356 and total tau in MOBSCs in a culture-phase-dependent manner

We next sought to evaluate pharmacological tools to lower p-tau Ser356 in live brain tissue. To determine whether response to NUAK inhibition differs under physiological conditions versus conditions of early Aβ dysregulation (which may initiate downstream tau changes), we generated mouse organotypic brain slice cultures (MOBSCs) from wildtype and APP/PS1 littermates. Previous work in MOBSCs has found that cultures undergo significant changes in the first 2 weeks in vitro, (including resolution of inflammation, increased neuronal outgrowth, developmental maturation and increased production of synaptic proteins [14, 24, 56]), before entering a relatively stable period. We, therefore, designed our experiments to capture both this initial period, and the following stable period.

WT or APP/PS1 MOBSCs were split into two culture phase groups (0–2 weeks in vitro, versus 2–4 weeks in vitro) and two treatment groups (DMSO or WZ4003) resulting in four experimental conditions represented in tissue from the same animal (Fig. 4a). In accordance with prior literature [37], we applied 5 µM WZ4003 to cultures and analysed the impact on proteins using Western blot (Fig. 4b). We explored whether 1) p-tau Ser356 levels change over time in culture (effect of phase), 2) whether early Aβ dysregulation results in changes to tau (effect of genotype), 3) whether WZ4003 treatment impacts levels of tau and 4) whether either genotype or age of the cultures impacts response to WZ4003 treatment. All conditions are graphically displayed normalised to the 0–2 week DMSO-treated condition from the same animal, whilst analysis was performed using ratio repeated measured analysis with the absolute data (Graphs of absolute data are in Supp. Fig. 3). For statistical analysis, the following LMEM was applied: *Protein level* ~ Genotype * Phase * Treatment + *(1|Litter/Animal).*

Regardless of genotype, we found that the levels of both total tau (Fig. 4c**,** effect of phase: *F_(1,51.00)_ = 6.28, *p* = 0.015) and p-tau Ser356 (Fig. 4d, effect of phase: **F_(1,51.00)_ = 8.73, *p* = 0.005) protein rise over time in culture. This likely reflects the regrowth or maturation of neurites in MOBSCs following the initial slicing procedure. The ratio of p-tauSer356/total tau remained stable over time in culture (Fig. 4e, effect of phase: *F*_(1,51.00)_ = 1.25, *p* = 0.27). In line with previous studies in neuroblastoma cells [37], application of WZ4003 resulted in a significant, and largely proportional, reduction in both total tau (Fig. 4c, effect of treatment: *F_(1,51.00)_ = 7.01, *p* = 0.011) and p-tau Ser356 (Fig. 4d**,** effect of treatment**:** **F_(1,51.00)_ = 10.7, *p* = 0.002) (Fig. 4e**,** effect of treatment on p-tau Ser356/total tau ratio: *F*_(1,51.00)_ = 2.10, *p* = 0.15). Interestingly, there was a trend interaction between culture phase and treatment for p-tau Ser356 (Fig. 4d, effect of treatment*phase: *F*_(1,51.00)_ = 2.83, *p* = 0.098), where the mean percentage loss of p-tau Ser356 between treated and control samples trended to be larger in the 0–2 week phase than the 2–4 week phase. The reduction in p-tau Ser356 averaged at 26.4% for WT and 26.6% for APP/PS1 0–2 week cultures, compared with 9.1% for WT and 6.8% for APP/PS1 2–4 week cultures.

In this study, we found no differences between genotypes in either baseline levels of tau, p-tau Ser356/total tau ratio, or in the degree of response to treatment (Fig. 4c–e). This shows that, in MOBSCs up to 4 weeks in vitro*,* the presence of mutations in the amyloid processing pathway does not result in perturbations to p-tau Ser356.

As we saw no differences between genotype, we performed an additional experiment in MOBSCs from WT mice to characterise WZ4003 impact on potential downstream phosphorylation sites of tau (Supp. Fig. 4). p-tau Ser202/Thr205 has previously been shown to be strongly associated with AD Braak stage [45] and phosphorylation of this site is sufficient to induce tau aggregation in vitro [15]. Preventing tau phosphorylation at Ser356 has also previously been found to prevent downstream phosphorylation of Ser202/Thr205 [46]. When examining the levels of p-tau Ser202/Thr205 in MOBSCs, we find a significant interaction between phase and treatment (*F_(1,18.00)_ = 5.09, *p* = 0.037), indicating that p-tau Ser202/Thr205 is lowered to a greater degree in 0–2 week cultures by WZ4003 than 2–4 week cultures (Supp. Fig. 4d, e). NUAK1 levels are not impacted by WZ4003 treatment (*F*_(1,18.00)_ = 0.82, *p* = 0.378) (Supp. Fig. 4 g, h). In this independent experiment, we also confirm the same effect of both phase (***F_(1,18)_ = 27.9, *p* < 0.001) and WZ4003 treatment (**F_(1,18.00)_ = 11.7, *p* = 0.003) on the levels of p-tau Ser356 (Supp. Fig. 4a, b), with a trend for a treatment*phase interaction (*F*_(1,18.00)_ = 3.45, *p* = 0.08).

### Synaptic and neuronal proteins rise over time in culture in MOBSCs, and are impacted by WZ4003 treatment

We next sought to examine the impact of WZ4003 treatment on both synaptic and neuronal protein levels in MOBSCs (Fig. 5). Here we tested whether the levels of PSD95 and Tuj1 are altered by: 1) age of the culture (effect of phase), 2) presence of APP/PS1 mutations (effect of genotype), 3) WZ4003 treatment and 4) whether genotype or culture age impacts response to treatment. For statistical analysis, the following LMEM was applied: *Protein level* ~ Genotype * Phase * Treatment + *(1|Litter/Animal).* Western blot analysis (Fig. 5a) revealed that the expression of the synaptic protein PSD95 increased over time in culture (Fig. 5b, effect of phase: ***F_(1,51.00)_ = 26.7, *p* < 0.001), but, interestingly, PSD95 was *reduced* in response to WZ4003 (Fig. 5b, effect of treatment: *F_(1,51.00)_ = 5.60, *p* = 0.022). The neuronal tubulin marker Tuj1 is similarly upregulated in 2–4 week cultures (Fig. 5c, effect of phase: ***F_(1,51.00)_ = 13.4, *p* < 0.001), but was not significantly impacted by WZ4003 treatment (Fig. 5c, effect of treatment: *F*_(1,51.00)_ = 1.99, *p* = 0.164). To examine the impact of synaptic proteins in the context of changes to neuronal proteins, we next normalised the levels of PSD95 to Tuj1 levels. Interestingly, the effect of phase remains (Fig. 5d, effect of phase: *F_(1,51.00)_ = 4.39, *p* = 0.041), indicating that synaptic proteins rise in excess of the rise in neuronal proteins over time, but the effect of treatment disappears (Fig. 5d**,** effect of treatment: *F*_(1,51.00)_ = 1.63, *p* = 0.207). This suggests that, despite the loss of Tuj1 in response to WZ4003 treatment not reaching significance alone, a WZ4003-induced loss of neuronal protein may partly contribute to the loss of PSD95. There were no differences between genotypes for either PSD95 or Tuj1 at baseline or in response to treatment (Fig. 5, Supp. Fig. 3).

When normalising total tau (Fig. 5e) or p-tau Ser356 (Fig. 5f), to Tuj1, we see that, relative to neuronal tubulin, there is a *reduction* in the levels of total tau (Fig, 5e, effect of phase: **F_(1,51.00)_ = 7.38, *p* = 0.009) and p-tau Ser356 (Fig. 5f, effect of phase: *F_(1,51.00)_ = 5.40, *p* = 0.024) in the 2–4 week cultures. This indicates, whilst there may be increased neuronal (Fig. 5c) and tau (Fig. 4c, d) protein over time, the *proportion* of tau relative to neuronal protein declines as the cultures age. Of particular note, is that the effect of WZ4003 treatment is abolished when normalising total tau (Fig. 5e, effect of treatment: *F*_(1,51.00)_ = 0.00, *p* = 0.964), or p-tau Ser356 (Fig. 5f, effect of treatment: *F*_(1,51.00)_ = 0.22, *p* = 0.642) to Tuj1, demonstrating that the lowering of tau, inhibition of NUAK, or other impacts of WZ4003 results in a proportional reduction in both tau and neuronal protein in MOBSCs up to 4 weeks in vitro.

### WZ4003 alters p-tau Ser356 levels in live human brain slice cultures

Finally, we sought to assess the impact of WZ4003 treatment in live adult human brain tissue. Healthy, peri-tumoral access tissue from five patients (demographics details listed in Table 5), was processed into 300 μm slices and cultured for 2 weeks in vitro (Fig. 6a). Cultures showed intact MAP2 positive neuronal cell bodies and processes at 14 *div* (Fig. 6b) and tau, neuronal and synaptic proteins were readily detectable by Western blot (Fig. 6c). p-tau Ser356 was detectable by Western blot in live human brain tissue prior to culturing (Supp. Fig. 5i, j). Slices from the same individuals were divided into both control (DMSO) and treatment (10 μM WZ4003) conditions, and protein levels were compared to the untreated control from the same patient. Graphs showing control-normalised data are used for display purposes (Fig. 6), with statistics run on ratio paired *T *tests using absolute data. Graphs of absolute data are shown in Supp. Fig. 5. For statistical analysis, the following LMEM was applied: *Protein level* ~ *Treatment* + *(1|CaseID).*

We found that WZ4003 treatment did not impact the level of total tau (Fig. 6d, mean % decrease = 2.2%, *t*_(4)_ = 0.43, *p* = 0.69), but there was a trend for reduced levels of p-tau Ser356 (Fig. 6e, mean % decrease = 35.0%, *t*_(4)_ = 2.43, *p* = 0.072) and a trend for reduced p-tau Ser356/tau ratio (Fig. 6f, mean % decrease = 31.6%, *t*_(4)_ = 2.17, *p* = 0.096). Interestingly, in contrast to the response in MOBSCs, we found a significant *increase* in the levels of the neuronal protein Tuj1 (Fig. 6g, mean % increase = 89.2%, *t_(4)_ = 3.39, *p* = 0.028). When normalising tau levels to Tuj1, we saw no significant loss of total tau (Fig. 6h, mean % decrease = 29.0%, *t*_(4)_ = 2.04, *p* = 0.11), but a significant loss of p-tau Ser356 (Fig. 6i, mean % decrease = 55.7%, **t_(4)_ = 4.81, *p* = 0.0086), indicating that WZ4003 treatment results in preferential lowering of p-tau Ser356 without incurring a loss of neuronal protein in HBSCs. We saw a trend for increased PSD95 in the WZ4003 treated cultures (Fig. 6j, mean % increase = 105%, *t*_(4)_ = 2.37, *p* = 0.077), that was likely proportional to the rise in Tuj1 levels (Fig. 6k, *t*_(4)_ = 0.739, *p* = 0.50). Overall, this demonstrates that WZ4003 treatment specifically reduces p-tau Ser356 in adult human brain tissue, relative to increases in neuronal and synaptic proteins.

## Discussion

This work combines multiple experimental tools to better characterise the timing and location of p-tau Ser356 in AD and assess the impact of pharmacological NUAK inhibition under a range of physiological and pathological conditions. Our work highlights p-tau Ser356 as a highly disease-associated form of tau in postmortem AD human brain. We show that p-tau Ser356 is not readily detectable in protein extracts from control postmortem brains, but can localise to dystrophic neurites surrounding areas of sporadic pathology in control tissue paraffin sections. We find an effect of Braak stage on the accumulation of p-tau Ser356, and potential downstream phosphorylation sites (p-tau Ser202/Thr205), in postmortem temporal lobe (BA20/21). In contrast to previous reports [37], we do not find an increase in NUAK1 levels in our AD brain cases, but we do find a subtle shift in NUAK1 protein expression into synaptoneurosome compartments in Braak III–IV and Braak VI samples. When examining paraffin sections from AD brain, we find that almost all (93%) of ThioS-positive tangles are dual-labeled with p-tau Ser356, indicating this epitope may be phosphorylated early in the tangle formation process. This finding is in agreement with studies in *Drosophila melanogaster* [2, 46] and neuroblastoma cells [37] suggesting that phosphorylation at this site can promote downstream phosphorylation of multiple other sites. Indeed, we find here that WZ4003 treatment also results in reduced levels of p-tau Ser202/Thr205 in MOBSCs. The consistent and early appearance of p-tau Ser356 in the AD disease course once again highlights this epitope as a potential therapeutic target. Interestingly a mutation in this site on the *MAPT* gene has been linked to a very-early onset form of FTD with Parkinsonism linked to chromosome 17 (FTDP-17) [44, 65, 67], so it seems likely that early changes to tau at Ser356 may be relevant across multiple dementia-causing diseases.

For the first time, we used array tomography, a microscopy method permitting sub-diffraction limit resolution characterisation of protein composition of individual synapses [33]*,* to assess whether p-tau Ser356 is present at the synapse in AD brains. We found that, whilst p-tau Ser356 is almost undetectable in control brain synapses, there is a small but significant proportion (~ 1 to 3%) of pre- and post-synapses that co-localise with p-tau Ser356 in AD brain with positive FRET signals indicating co-localisation of p-tau356 with either synaptophysin or PSD95 within the synaptic compartments. Interestingly, whilst a similar proportion of synapses co-localise with AT8 (p-tau Ser202/Thr205), the proportion of synapses that contain both epitopes is considerably lower (~ 0 to 1%), raising the possibility that the order of tau phosphorylation may be different in individual synapses. Alternatively, it may be that detection of both epitopes together is under-represented through technical limitations, such as reduced antibody binding when both epitopes co-localise. Building on recent reports that astrocytes engulf synapses in AD brain [59, 62], we find that p-tau Ser356-containing pre-synapses are around five times more likely to be ingested by astrocytes than the general pre-synapse population, potentially highlighting a role in targeting synapses for phagocytosis. Recent work has highlighted potentially important roles of synaptic tau for both toxicity and involvement of trans-synaptic tau spread [9, 36, 47, 68]. Future work exploring the impact of synaptic p-tau Ser356, in contrast to tau phosphorylated at alternative sites, could further elucidate its role in AD pathology.

Given the potential importance of NUAK1 in the phosphorylation of tau at Ser356, and our findings that p-tau Ser356 is highly associated with disease progression in AD, we sought to characterise the impacts of pharmacological NUAK inhibition under a range of physiological and disease-model conditions. In this study, we used WZ4003, which has previously been shown to be a potent inhibitor of NUAK1, and to a lesser extent NUAK2, and with no inhibitory activity on a panel of 139 other related kinases [5]. Previous studies using WZ4003 have used simple in vitro systems such as primary culture [8] or cell lines [5, 37] which may oversimplify the impacts of NUAK inhibition on brain tissue containing multiple cell types, and functionally relevant neuronal architecture [19]. In addition, prior work looking at the effect of NUAK1 knockdown in animal models (*Drosophila melanogaster* and mouse) focussed on models with tau pathology exclusively, leaving a gap in our understanding of how Aβ pathway dysregulation, or elevated Aβ production, may impact response to NUAK inhibition [37]. Here, we used MOBSCs from the APP/PS1 mouse model of Aβ pathology, alongside wildtype littermates, to model first, whether we see changes to p-tau Ser356 expression in this AD model, and then the implications of targeting NUAK activity, using WZ4003, under physiological (wildtype) or Aβ pathology (APP/PS1) conditions. Whilst previous work has found that MOBSCs can show accelerated pathological changes compared to in vivo [12, 13, 20, 28]*,* and APP/PS1 mice are found to show increased tau phosphorylation with age [43], we did not find any differences between the genotypes in this study up to 4 weeks in culture. Nevertheless, the APP/PS1 cultures served an important purpose to establish whether conditions of elevated Aβ production alter biological responses to WZ4003 treatment. In our work, the response of APP/PS1 MOBSCs was not statistically different to their WT littermates.

A unique aspect of this study is the use of both MOBSCs and HBSCs to examine the impact of WZ4003 treatment. In MOBSCs, we found that, whilst WZ4003 treatment lowered both total tau and p-tau Ser356 protein, this reduction coincided with a loss of PSD95, and was proportional to a loss of the neuronal tubulin marker Tuj1. Interestingly, the effects of WZ4003 treatment on tau, synaptic and neuronal protein levels were strongest in the 0–2 week culture period, indicating the early stage cultures were especially sensitive to negative impacts of NUAK inhibition, loss of tau protein or any potential off-target effects of WZ4003. This possibly reflects differential involvement of NUAK1/2, altered processing of tau, or differential vulnerability to off-target (non-tau) effects of WZ4003/NUAK inhibition in different culture phases. By contrast, WZ4003 treatment in HBSCs resulted in a specific reduction in p-tau Ser356, whilst preserving total tau, that occurred alongside *increased* levels of neuronal and synaptic protein. These findings could demonstrate important species differences in how NUAK1 regulates tau and highlight the benefits of using human experimental systems to assess impacts of pharmacological agents [6, 53, 61]. However, another key difference between MOBSCs and HBSCs is the age of the brain tissue used to generate slices. MOBSCs are taken from postnatal (P6–P9) animals, whilst the age of human brain in this study ranged from 37 to 76 years old. Therefore, another interpretation of the different response to WZ4003 in MOBSCs versus HBSCs is potential differences in the role of NUAK1/2 during development versus ageing [7]. Indeed, a number of studies have identified key roles of NUAK1 in regulating a number of developmental processes including axon elongation [8, 10, 11], axon branching [10], and cortical development [11]. It may be that the postnatal slice cultures are negatively affected by either NUAK1/2 inhibition or the loss of total tau during this period, whilst adult human tissue is less dependent on NUAK activity (or benefits from the relative preservation of total tau levels, which may be important for physiological function [34]). Indeed, our results here indicate NUAK1 inhibition may *increase* neuronal and synaptic protein levels in adult human brain tissue.

Human slice cultures represent a translationally powerful new tool for neuroscience research [1, 6, 22, 38, 41, 42, 50, 51, 53, 54, 61]. Although live human tissue has historically been difficult to obtain, with close collaboration with neurosurgery units, research nurse teams and the laboratory scientists, we have established an efficient pipeline to obtain and culture human brain slices. We show here that they can be an effective tool to examine the impact of pharmacological compounds in live human brain tissue. Previous work has shown benefits of using human cerebrospinal fluid to boost longevity of HBSCs, particularly in regards to electrophysiological activity [53, 54]. In our work, using an enriched stem-cell-like medium [41], we find HBSCs retain MAP2 positive neuronal cell bodies and intact neurites, and we are readily able to detect tau, neuronal and synaptic proteins via Western blot for at least 2 weeks in vitro. By comparing control and treatment conditions in slices taken from the same individual, we are able to detect biologically relevant responses, even on a background of unavoidable variations in patient age, sex, brain region taken and variations in patient lifestyle and genetic factors. It is worthy of comment that all of our HBSCs, despite none being clinically diagnosed with AD, had detectable levels of p-tau Ser356 in protein extracts, in contrast to our postmortem study which found very little p-tau Ser356 in Braak 0–I control protein extracts. As we are able to detect p-tau Ser356 in acute samples taken from surgery, prior to culturing, this could indicate that p-tau is susceptible to degradation in the post-mortem interval, and thus small levels of p-tau in control postmortem brain in our samples were rendered undetectable. Alternatively, this could represent rapid increases in tau phosphorylation in response to damage during brain surgery [32, 34], exposure to anaesthetic [23, 49], or fundamental differences between peri-tumour and post-mortem “control” tissue. We should also be mindful that whilst the tissue itself is non-tumour, we cannot rule out alterations to NUAK1 expression or activity in these samples, which have originated from individuals with brain tumours [39]. Such differences will be important to reflect on as the tool becomes more widely used. The use of HBSCs as a research tool is expanding and comparison between mouse tissue, primary cultures, postmortem human and live human tissue models is likely to be highly valuable when assessing the translational viability of future therapies under development.

One limitation of the present study is that it uses a single small molecule tool which has activity at both NUAK1 and NUAK2, and whilst the published kinase-selectivity data suggest it is a relatively clean inhibitor [5], we cannot rule out activities at other kinases. Future work should explore further compounds with selectivity for NUAK1 over NUAK2 and more complete profiles. We should also consider that NUAK1 also acts on other, non-tau targets, such as the TGF-β pathway, so cannot rule out that some of the impacts we see on synaptic/neuronal proteins may be due to alterations in NUAK function beyond phosphorylating tau [7]. Future work to develop specific tools to target p-tau Ser356 directly, such as humanised antibodies, could be highly informative.

In summary, the work in this study further highlights p-tau Ser356 as a potential target of interest in developing AD therapeutics, with increased p-tau Ser356 strongly correlating with Braak stage, being a near-ubiquitous presence in NFTs and co-localising with synapses in AD brain. Whilst NUAK inhibition via WZ4003 treatment of postnatal MOBSCs results in tau lowering that is proportional to loss of synaptic and neuronal protein, we find WZ4003 effectively and specifically lowers p-tau Ser356 in live, adult human brain tissue, highlighting the importance of using complementary experimental systems in pre-clinical work. Future work should further explore the impact of pharmacological NUAK inhibition in vivo using a range of tau and Aβ pathology models and with NUAK1 inhibitors optimised for increased potency and drug-like properties with more fully characterised selectivity profiles.

## Supplementary Information

Below is the link to the electronic supplementary material.

Supplementary file1 (PDF 1075 KB)

## Data Availability

Data are deposited on the University of Edinburgh DataShare digital repository or are available at reasonable request from the corresponding author.
