# Supplementary material for: p-tau Ser356 is associated with Alzheimer’s disease pathology and is lowered in brain slice cultures using the NUAK inhibitor WZ4003
Source: Acta Neuropathol. 2024 Jan 4;147(1):7. doi: 10.1007/s00401-023-02667-w (PMC10766794; doi:10.1007/s00401-023-02667-w)
Supplement: Supplementary file 1 — Supplementary file1 (PDF 1075 KB) [file 401_2023_2667_MOESM1_ESM.pdf]

Supplementary Methods

FRET Imaging

Using tissue from AD patients, Förster resonance energy transfer (FRET) images were taken to allow for the sensitive detection of interactions between fluorophores <10nm apart. Here, array tomography ribbons were immunostained with two different antibody combinations to examine FRET between p-tau Ser356 and pre- or post-synapses (Supplementary Table 1). Images of consecutive sections of the same field of view were taken using a Leica TCS8 confocal with a 63x 1.4 NA oil objective. Alexa fluor 405, 488, and 568/Cy3 were excited sequentially and imaged. For FRET analysis, the spectral window of 568/Cy3 (the acceptor, 570 to 634 nm) was also imaged under the excitation of 488 nm (the donor), thus recording the energy transfer from donor molecules to acceptors based on intensity (emission FRET[69]). Laser and detector settings were set at the beginning of each imaging session on a positive control to avoid saturation. For FRET analysis, donor-only (488) and acceptor-only (568 or Cy3) samples were prepared and imaged to calculate the donor emission crosstalk with the acceptor emission (beta parameter) and the direct excitation of the acceptor by the donor excitation laser line (gamma parameter)[69]. Positive control samples labelling the same protein with both donor and acceptor fluorophores with secondary and tertiary antibodies were stained and imaged to ensure FRET signals could be detected in each experiment. Images were aligned and thresholded using in-house Fiji (ImageJ) and MATLAB scripts. Stacks of aligned images corresponding to the acceptor emission under donor excitation (referred to as the FRET image) were corrected for beta and gamma parameters. Thresholded image masks corresponding to the donor (postsynaptic terminals or presynapses) and the acceptor (p-tau Ser356) images were used to identify areas of overlap in the corrected FRET image. These areas were used to quantify the percentage of pixels exhibiting any FRET signal, providing a qualitative measure of the presence of the FRET effect. The resulting parameter data was statistically analysed using custom R Studio scripts. Analysis code is available on GitHub (https://github.com/Spires-Jones-Lab).

	Primary Antibody	Source	Catalogue Number	Secondary Antibody	Source	Catalogue Number
Stain 1	1:200 chicken GFAP	Abcam	ab4674-50ul	1:50 goat anti-chicken Alexa-fluor 405	Invitrogen	A48260
	1:500 Rabbit p-tau Ser356	Abcam	ab75603	1:50 donkey anti-rabbit Alexa-fluor 568	Abcam	ab175470
	1:100 mouse synaptophysin	Abcam	ab8049	1:50 goat anti-mouse Alexa-fluor 488	Invitrogen	A21121
Acceptor Only 1	1:200 chicken GFAP	Abcam	ab4674-50ul	1:50 goat anti-chicken Alexa-fluor 405	Invitrogen	A48260
	1:500 Rabbit p-tau Ser356	Abcam	ab75603	1:50 donkey anti-rabbit Alexa-fluor 568	Abcam	ab175470
Donor Only 1	1:200 chicken GFAP	Abcam	ab4674-50ul	1:50 goat anti-chicken Alexa-fluor 405	Invitrogen	A48260
	1:100 mouse synaptophysin	Abcam	ab8049	1:50 goat anti-mouse Alexa-fluor 488	Invitrogen	A21121
Positive Control 1	1:100 mouse synaptophysin	Abcam	ab8049	1:50 goat anti-mouse Alexa-fluor 488	Invitrogen	A21121
				1:50 donkey anti-goat Alexa-fluor 568	Abcam	ab175474
Stain 2	1:200 chicken GFAP	Abcam	ab4674-50ul	1:50 goat anti-chicken Alexa fluor-405	Invitrogen	A48260
	1:500 Rabbit p-tau Ser356	Abcam	ab75603	1:50 goat anti-rabbit Cy3	Abcam	ab6939
	1:200 guinea pig PSD95	Synaptic Systems	124 014	1:50 goat anti-guinea pig Alexa fluor-488	Abcam	ab150185
Acceptor Only 2	1:200 chicken GFAP	Abcam	ab4674-50ul	1:50 goat anti-chicken Alexa fluor-405	Invitrogen	A48260
	1:500 Rabbit p-tau Ser356	Abcam	ab75603	1:50 goat anti-rabbit Cy3	Abcam	ab6939
Donor Only 2	1:200 chicken GFAP	Abcam	ab4674-50ul	1:50 goat anti-chicken Alexa fluor-405	Invitrogen	A48260

	1:200 guinea pig PSD95	Synaptic Systems	124 014	1:50 goat anti-guinea pig Alexa fluor-488	Abcam	ab150185
Positive Control 2	1:200 guinea pig PSD95	Synaptic Systems	124 014	1:50 goat anti-guinea pig Alexa fluor-488	Abcam	ab150185
				1:50 donkey anti-goat Alexa-fluor 568	Abcam	ab175474

Supplementary Table 1: *Summary table for primary and secondary antibody combinations used in FRET experiments (**Supp. Fig. 2**).*

BBN	SD	Clinical Diagnosis	Braak Stage	Thal Phase	Age (yrs)	Sex	PMI (hrs)	Lewy Body?	Study Used
001.24527	SD056/14	AD	V	5	81	M	74	N	Supp. Fig 2a-c
001.28771	SD010/16	AD	VI	5	85	M	91	N	Supp. Fig 2a-c
001.32929	SD012/18	AD	VI	5	85	F	80	N	Supp. Fig 2a-c
001.25739	SD014/15	AD	VI	5	85	F	45	N	Supp. Fig 2a-c
001.26718	SD040/15	AD	VI	5	78	M	74	N	Supp. Fig 2a-c
001.24322	SD049/14	AD	VI	5	80	M	101	N	Supp. Fig 2d-f
001.26718	SD040/15	AD	VI	5	78	M	74	N	Supp. Fig 2d-f
001.32929	SD012/18	AD	VI	5	85	F	80	N	Supp. Fig 2d-f
001.29695	SD004/17	AD	VI	5	86	M	72	Y Limbic	Supp. Fig 2d-f
001.25739	SD014/15	AD	VI	5	85	F	45	N	Supp. Fig 2d-f
001.30973	SD039/17	AD	VI	5	89	F	96	N	Supp. Fig 2d-f

Supplementary Table 2: *Summary demographic and neuropathological characteristics of human post-mortem subjects used in FRET experiments (**Supp. Fig. 2**). AD = Alzheimer's disease, BBN = Medical Research Council Brain Bank Number, SD = Edinburgh Brain Bank Number, PMI = Post-Mortem Interval.*

Supplementary Figures

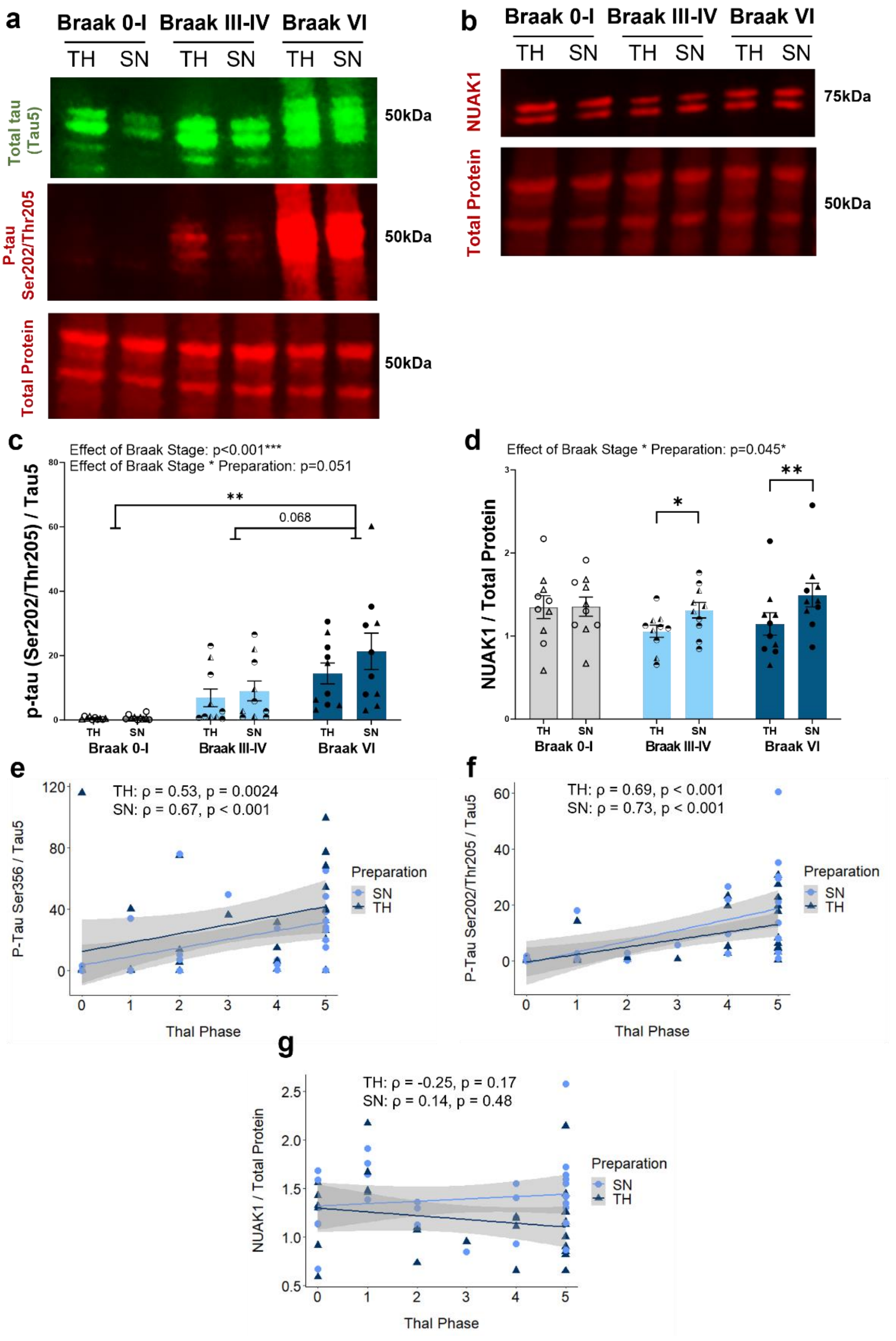

Supplementary Figure 1: Additional characterisation of p-tau and NUAK changes with Braak stage and Thal phase in post-mortem human brain. (a, b) *Representative Western blot images of total homogenates (TH) and synaptoneurosomes (SN) from Braak 0-I, III-IV or VI (diagnosed AD) stage post-mortem brain. Blots were probed with total protein (REVERT stain)* ***(a, b)****, total tau (Tau5)* ***(a)****, p-*

tau Ser202/Thr205 ***(a)*** *and NUAK1* ***(b).*** ***(c)*** *There is a significant increase in p-tau Ser202/Thr205 (normalised to total tau) with increasing Braak stage (****$F_{(2,32.12)}$*=10.7, p<0.001), with a significant difference between Braak 0-I and Braak VI AD (***$t_{(27)}$*=4.05, p=0.0011). There is a non-significant trend for an interaction between Braak stage and preparation for p-tau Ser202/Thr205 (normalised to total tau) (*$F_{(2,27.00)}$*=3.32, p=0.051).* ***(d)*** *There is no significant effect of Braak stage (*$F_{(2,36.83)}$*=0.64, p=0.531) or preparation (TH vs SN) (*$F_{(1,27.00)}$*=0.00, p=0.951) on NUAK1 levels in human post-mortem brain.* ***(d)*** *There is a significant interaction between Braak stage and preparation (**$F_{(2,27.00)}$*=3.48, p=0.045), with a significant increase in NUAK1 expression in synaptoneurosomes vs total homogenates in Braak III-IV (**$t_{(27)}$*=2.68, p=0.0124) and VI (***$t_{(27)}$*=3.68, p=0.001) stage tissue. N = 10 cases per Braak stage.* ***(e)*** *There is a significant positive correlation between the p-tau Ser356 / Tau5 ratio and Thal phase for TH (ρ=0.53, p=0.0024, 95% CI: [-0.0025,0.64]) and SN (ρ=0.67, p<0.001, 95% CI: [-0.17,0.73]).* ***(f)*** *There is a significant positive correlation between the p-tau Ser202/Thr205 / Tau5 ratio and Thal phase for TH (ρ=0.69, p<0.001, 95% CI: [0.27,0.78]) and SN (ρ=0.73, p<0.001, 95% CI: [0.25,0.76]).* ***(g)*** *There is a non-significant negative correlation between NUAK1 and Thal phase for TH (ρ=-0.25, p=0.17, 95% CI: [-0.53,0.16]), and a non-significant positive correlation for SN (ρ=0.14, p=0.48, 95% CI: [-0.24,0.47]). Each point on the graphs represents a single case. For* ***(c, d)*** *triangles = males, circles = females. For* ***(e, f, g)*** *triangles = TH, circles = SN.*

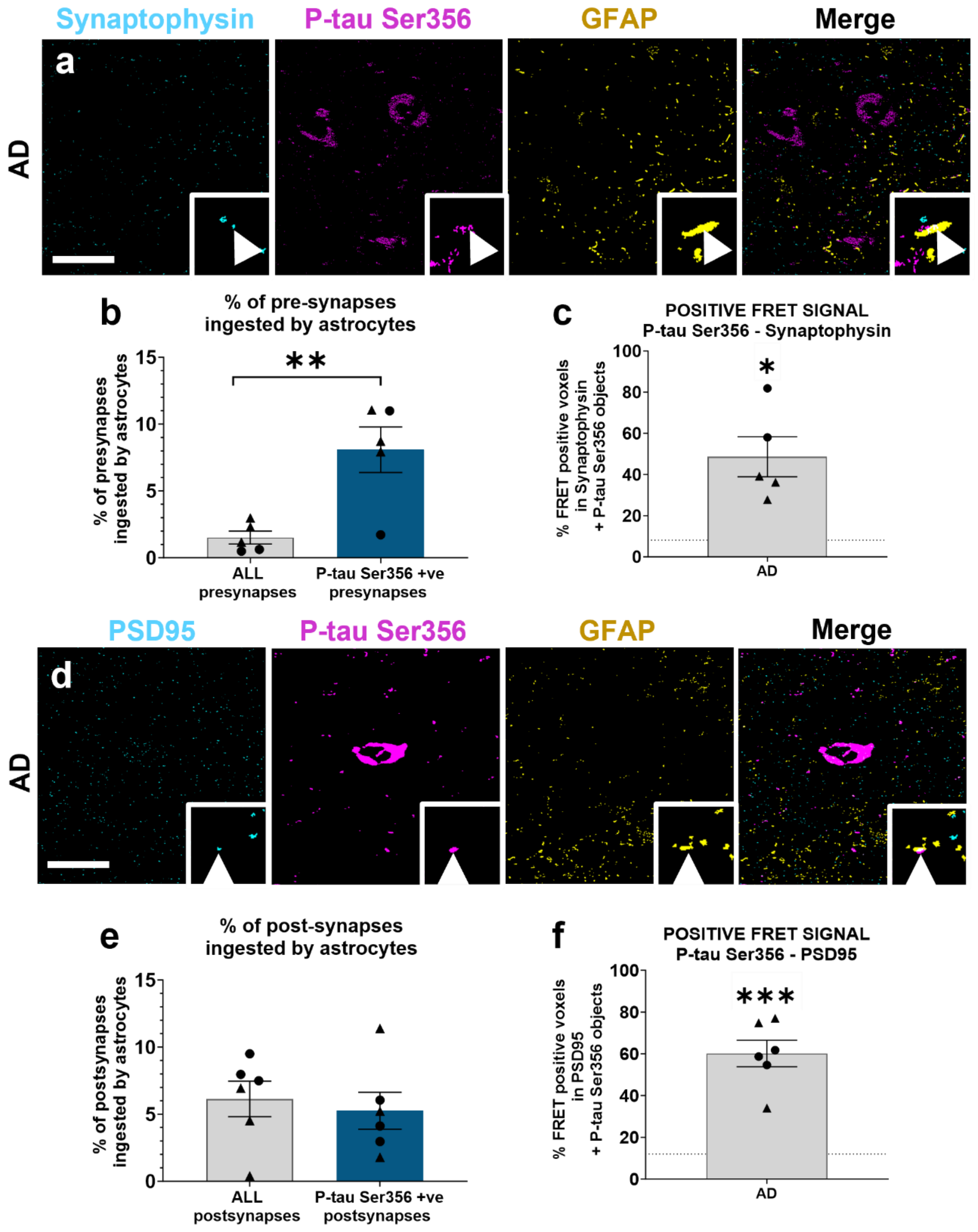

Supplementary Figure 2: P-tau Ser356 co-localises with pre- and post-synapses inside astrocytes in Alzheimer's disease post-mortem brain and shows a positive FRET signal with synaptophysin and PSD-95. (a, d) *Representative images of 70nm thick array tomography images from AD post-mortem brain. Sections were stained with the presynaptic marker synaptophysin (cyan)* ***(a)****, postsynaptic marker PSD-95* ***(d)****, p-tau Ser356 (magenta), GFAP (yellow). The white arrow indicates the area of co-localisation of synaptophysin or PSD-95, p-tau Ser356 and GFAP. Scale bar represents 20 µm, square insets represent 5 µm x 5 µm. There is a significant increase in synaptophysin co-localising with GFAP in p-tau Ser356-positive pre-synapses, compared with all pre-synapses regardless of tau status, in AD brain (**$F_{(1,14)}$=9.94, p=0.00705)* ***(b)****. There is a significant increase in positive FRET signal between p-tau Ser356 and synaptophysin in AD brain relative to donor-only negative control (*$t_{(4)}$=4.18,*

p=0.014) ***(c)****. There is no significant difference in PSD-95 co-localising with GFAP in p-tau Ser356-positive postsynapses relative to all postsynapses in AD brain ($F_{(1,17)}$=0.0341, p=0.856)* ***(e)****. There is a significant increase in positive FRET signal between p-tau Ser356 and PSD-95 in AD brain relative to donor-only negative control (***$t_{(5)}$=7.56, p=0.0006)* ***(f)****. Each point on the graph represents a single case, males = triangles, females = circles. N = 5 AD cases for* ***(a-c)*** *and N = 6 AD cases for* ***(d-f).***

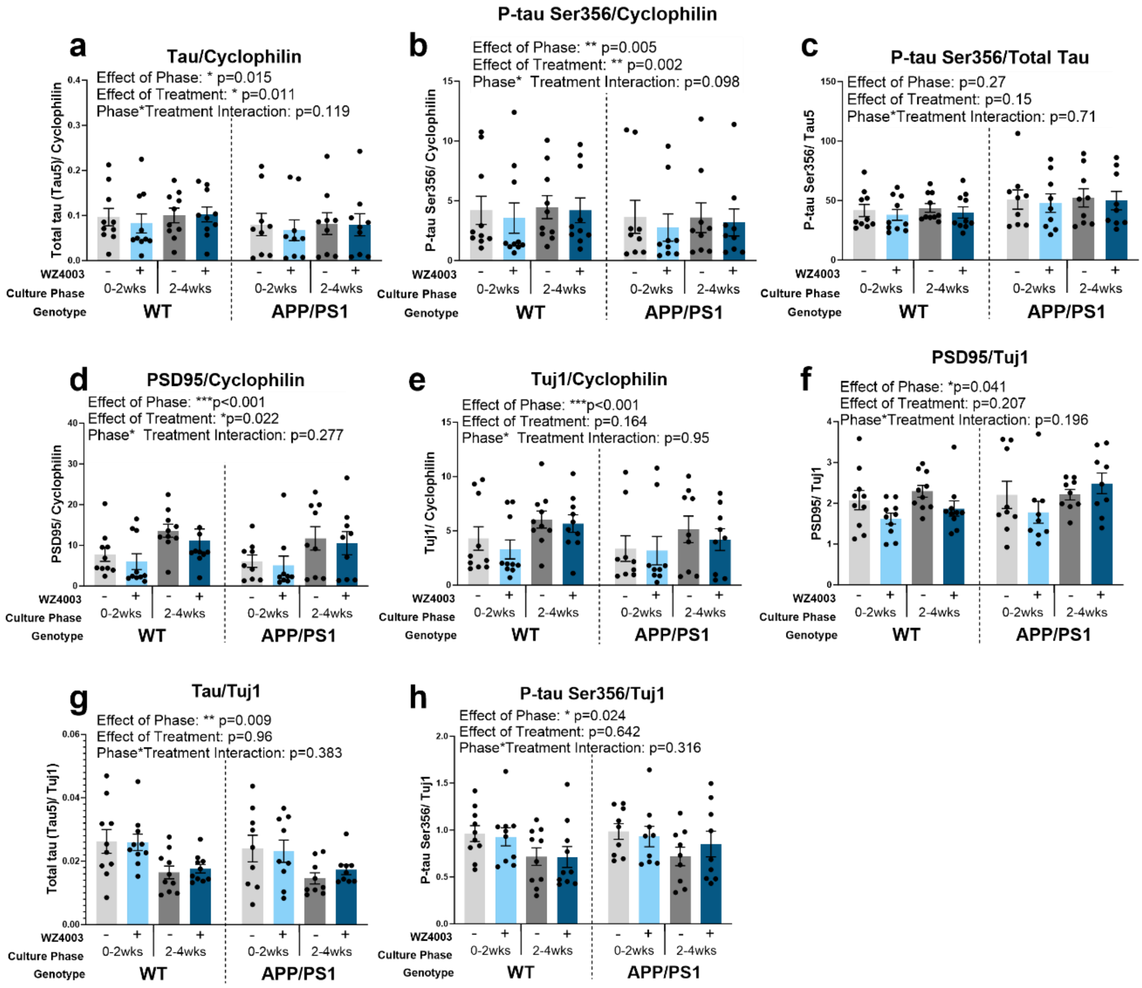

Supplementary Figure 3: Absolute data graph display for mouse brain slice culture western blots. *Data and statistics are the same as in Figure 4 and 5, but graphically displayed to show absolute values across mice, not normalised to 0-2 week control to highlight the lack of genotype effects.* ***(a)*** *There is a significant effect of phase (*$F_{(1,51.00)}$=6.28, p=0.015) and treatment (*$F_{(1,51.00)}$=7.01, p=0.011) on the levels of total tau, but no effect of genotype ($F_{(1,21.30)}$=0.02, p=0.894).* ***(b)*** *There is a significant effect of phase (**$F_{(1,51.00)}$=8.73, p=0.005) and treatment (**$F_{(1,51.00)}$=10.7, p=0.002) on the levels of p-tau Ser356, and a trend interaction between Phase*Treatment ($F_{(1,51.00)}$=2.83, p=0.098), but there is no effect of genotype ($F_{(1,26.41)}$=0.00, p=0.997).* ***(c)*** *There are no significant effects of phase ($F_{(1,51.00)}$=1.25, p=0.27), treatment ($F_{(1,51.00)}$=2.10, p=0.15) or genotype ($F_{(1,26.51)}$=0.08, p=0.781) on the p-tau Ser356/total tau ratio.* ***(d)*** *There is a significant effect of phase (***$F_{(1,51.00)}$=26.7, p<0.001) and treatment (*$F_{(1,51.00)}$=5.60, p=0.022) on the levels of PSD95 normalised to cyclophilin, but no effect of genotype ($F_{(1,41.92)}$=0.21, p=0.652).* ***(e)*** *There is a significant effect of phase (***$F_{(1,51.00)}$=13.4, p<0.001) on the levels of Tuj1 normalised to cyclophilin, but no effects of treatment ($F_{(1,51.00)}$=1.99, p=0.164) or genotype ($F_{(1,41.92)}$=0.00, p=0.957).* ***(f)*** *There is a significant effect of phase (*$F_{(1,51.00)}$=4.39, p=0.041) but no effect of treatment ($F_{(1,51.00)}$=1.63, p=0.207) or genotype*

($F_{(1,47.89)}$=0.13, p=0.715) on the levels of PSD95 when normalised to Tuj1. **(g)** There is a significant effect of phase (**$F_{(1,51.00)}$=7.38, p=0.009), but no effect of treatment ($F_{(1,51.00)}$=0.00, p=0.964) or genotype ($F_{(1,42.38)}$=0.08, p=0.774), on the levels of total tau when normalised to Tuj1. **(h)** There is a significant effect of phase (*$F_{(1,51.00)}$=5.40, p=0.024), but no effect of treatment ($F_{(1,51.00)}$=0.22, p=0.642) or genotype ($F_{(1,42.83)}$=0.00, p=0.957), on the levels of p-tau Ser356 when normalised to Tuj1. N = 9 APP/PS1 and 10 WT animals, 1-2 slices per animal per condition.*

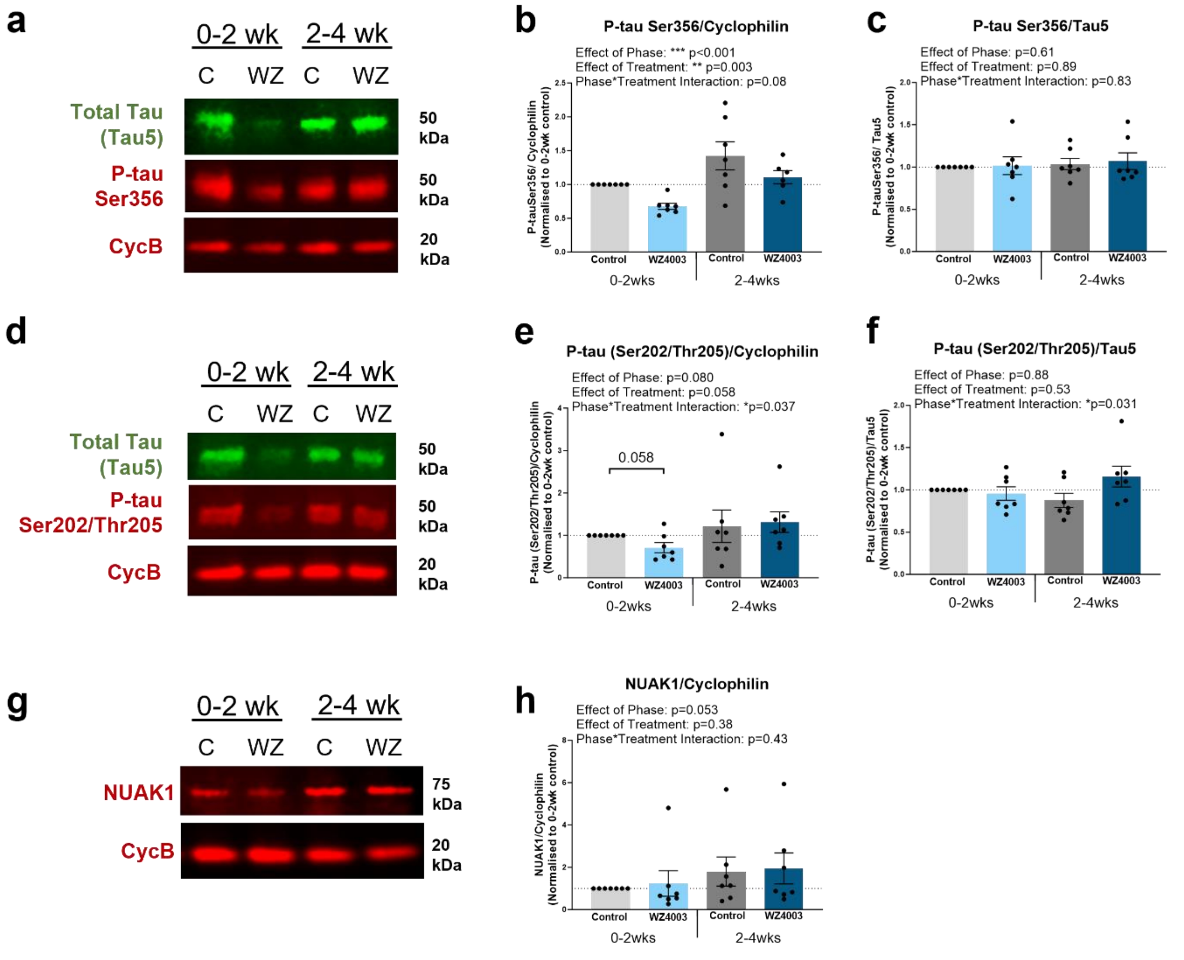

Supplementary Figure 4: Additional characterisation of changes to tau phosphorylation and NUAK1 levels in WT MOBSCs. ***(a, d, g)*** *Representative Western blot images for WT MOBSCs probed for total tau* ***(a, d)****, p-tau Ser356* ***(a)****, p-tau Ser202/Thr205* ***(d)****, NUAK1* ***(g)*** *and housekeeping protein cyclophilin* ***(a, d, g)****. The following graphs are displayed normalised to 0-2 week control for each animal to show relative differences, but statistics are performed on absolute data.* ***(b)*** *There is a significant effect of phase (***$F_{(1,18)}$=27.9, p<0.001) and treatment (**$F_{(1,18.00)}$=11.7, p=0.003) on the levels of p-tau Ser356, and a trend interaction between Phase*Treatment ($F_{(1,18.00)}$=3.45, p=0.08).* ***(c)*** *There are no significant effects of phase ($F_{(1,18.00)}$=0.26, p=0.614) or treatment ($F_{(1,18.00)}$=0.02, p=0.890) on the p-tau Ser356/total tau ratio.* ***(e)*** *There is a significant interaction between Phase*Treatment (*$F_{(1,18.00)}$=5.09, p=0.037), and trend effects of phase ($F_{(1,18.00)}$=3.45, p=0.08) and treatment ($F_{(1,18.00)}$=4.09, p=0.058) on the levels of p-tau Ser202/Thr205.* ***(F)*** *There is a significant interaction between Phase*Treatment (*$F_{(1,18.00)}$=5.45, p=0.031), and no effects of phase ($F_{(1,18.00)}$=0.02, p=0.880) or treatment ($F_{(1,18.00)}$=0.41, p=0.532) on the p-tau (Ser202/Thr205)/total tau ratio.* ***(h)*** *There is a trend effect of*

phase ($F_{(1,18.00)}$=4.29, p=0.053), but no effect of treatment ($F_{(1,18.00)}$=0.82, p=0.378) on NUAK1 levels. N = 7 animals, 1-2 slices per animal per condition.

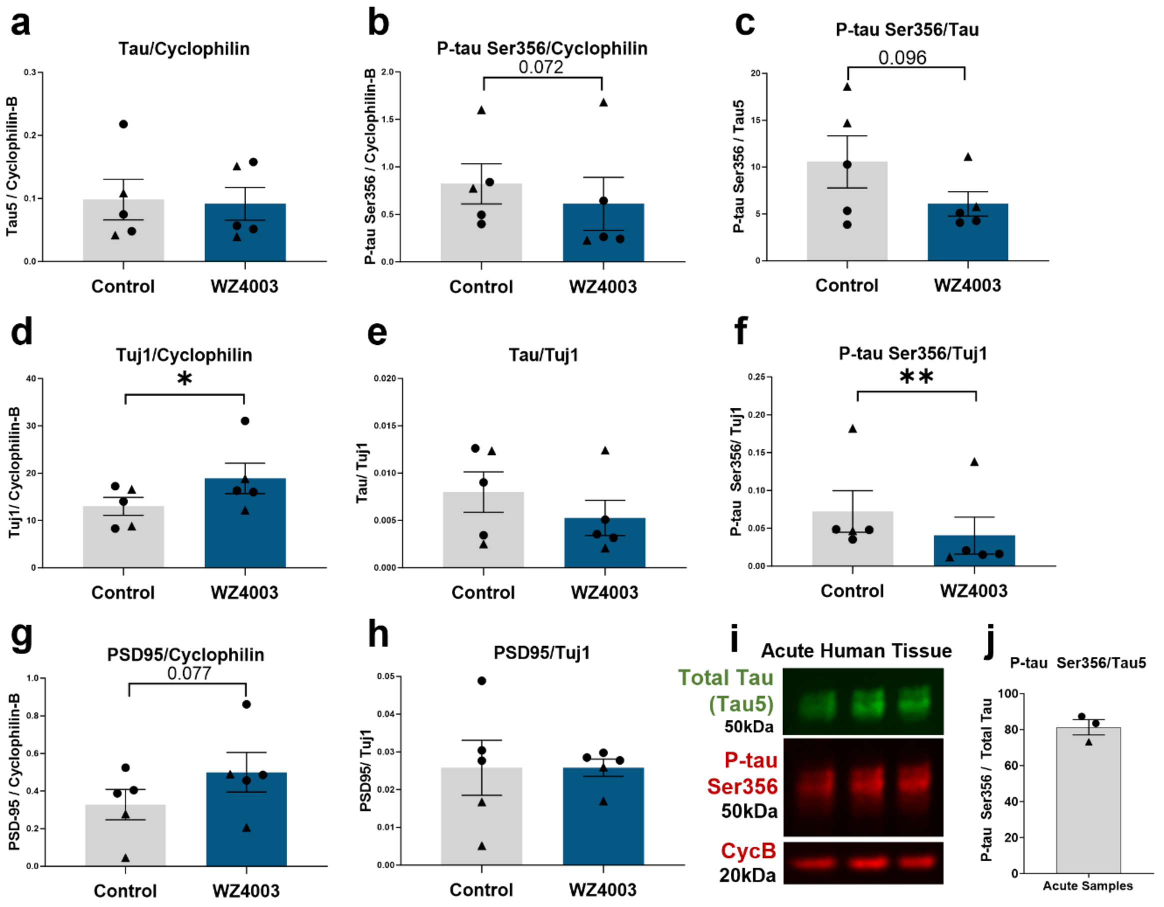

Supplementary figure 5: Absolute data graph display for human brain slice culture western blots. *Data and statistics are the same as in Figure 6, but graphically displayed to show absolute values across cases, not normalised to control.* ***(a)*** *WZ4003 does not significantly alter levels of tau (normalised to cyclophilin) ($t_{(4)}$=0.43, p=0.688).* ***(b)*** *There is a trend for WZ4003 treatment to reduce p-tau Ser356 (normalised to cyclophilin) ($t_{(4)}$=2.43, p=0.072).* ***(c)*** *There is a trend for WZ4003 to reduce the ratio of p-tau Ser356 / total tau ($t_{(4)}$=2.17, p=0.096).* ***(d)*** *WZ4003 treatment significantly increased Tuj1 levels (*$t_{(4)}$=3.39, p=0.028).* ***(e)*** *WZ4003 does not significantly alter tau levels as a proportion of neuronal protein ($t_{(4)}$=2.04, p=0.11).* ***(f)*** *WZ4003 significantly lowers p-tau Ser356 as a proportion of neuronal protein (**$t_{(4)}$=4.81, p=0.0086).* ***(g)*** *There is a trend for WZ4003 to increase PSD95 protein (normalised to cyclophilin) ($t_{(4)}$=2.37, p=0.077).* ***(h)*** *There is no effect of WZ4003 on PSD95 protein (normalised to Tuj1) ($t_{(4)}$=0.74, p=0.501).* ***(i-j)*** *P-tau Ser356 is readily detectable in acute cortical access tissue used to generate human brain slice cultures (N = 3). N = 5 cases per condition. Each point represents an individual human case, triangles = males, circles = females.*
